# Assessing the household economic burden of non-communicable diseases in India: evidence from repeated cross-sectional surveys

**DOI:** 10.1186/s12889-021-10828-3

**Published:** 2021-05-07

**Authors:** Veenapani Rajeev Verma, Piyush Kumar, Umakant Dash

**Affiliations:** Department of Humanities and Social Sciences, Indian Institute of Technology Madras, Chennai, India

**Keywords:** Catastrophic health expenditure, Non communicable diseases, Impoverishment

## Abstract

**Background:**

Financing for NCDs is encumbered by out-of-pocket expenditure (OOPE) assuming catastrophic proportions. Therefore, it is imperative to investigate the extent of catastrophic health expenditure (CHE) on NCDs, which are burgeoning in India. Thus, our paper aims to examine the extent of CHE and impoverishment in India, in conjunction with socio-economic determinants impacting the CHE.

**Methods:**

We used cross-sectional data from nationwide healthcare surveys conducted in 2014 and 2017–18. OOPE on both outpatient and inpatient treatment was coalesced to estimate CHE on NCDs. *Incidence* of CHE was defined as proportion of households with OOPE exceeding 10% of household expenditure. *Intensity* of catastrophe was ascertained by the measure of *Overshoot* and *Mean Positive Overshoot Indices*. Further, impoverishing effects of OOPE were assessed by computing *Poverty Headcount Ratio* and *Poverty Gap Index* using India’s official poverty line. Concomitantly, we estimated the inequality in incidence and intensity of catastrophic payments using *Concentration Indices*. Additionally, we delineated the factors associated with catastrophic expenditure using *Multinomial Logistic Regression.*

**Results:**

Results indicated enormous incidence of CHE with around two-third households with NCDs facing CHE. Incidence of CHE was concentrated amongst poor that further extended from 2014(CI = − 0.027) to 2017–18(CI = − 0.065). Intensity of CHE was colossal as households spent 42.8 and 34.9% beyond threshold in 2014 and 2017-18 respectively with poor enduring greater overshoot vis-à-vis rich (CI = − 0.18 in 2014 and CI = − 0.23 in 2017–18). Significant immiserating impact of NCDs was unraveled as one-twelfth in 2014 and one-eighth households in 2017–18 with NCD burden were pushed to poverty with poverty deepening effect to the magnitude of 27.7 and 30.1% among those already below poverty on account of NCDs in 2014 and 2017–18 respectively. Further, large inter-state heterogeneities in extent of CHE and impoverishment were found and multivariate analysis indicated absence of insurance cover, visiting private providers, residing in rural areas and belonging to poorest expenditure quintile were associated with increased likelihood of incurring CHE.

**Conclusion:**

Substantial proportion of households face CHE and subsequent impoverishment due to NCD related expenses. Concerted efforts are required to augment the financial risk protection to the households, especially in regions with higher burden of NCDs.

**Supplementary Information:**

The online version contains supplementary material available at 10.1186/s12889-021-10828-3.

## Introduction

Non-communicable diseases (NCDs) has assumed significant proportions in contributing to the overall disease burden, measured in disability-adjusted life years (DALYs) in India, over the past 30 years [1]. India’s State-Level Disease Burden Initiative affirmed that every state in India is currently grappling with higher burden of NCDs and injuries vis-à-vis infectious diseases. In 2017, NCDs accounted for 63.7% of all mortality and was a major contributor in the cost of treatment for inpatient admissions (40%) and ambulatory care (35%). While India witnessed a paradigm shift in its commitment towards Universal Health Coverage post 2014, the promise of universality would be delivered only if its structure and implementation recognize and respond to NCD related poverty [2].

The unprecedented financial burden posed by NCDs is two- pronged; *Firstly,* at the macroeconomic level, burden posed by NCDs has deleterious effects on economic growth of nations. A 2011 report delving on economic burden of NCDs in low-and-middle income countries concluded that major NCDs like cardiovascular diseases, cancer and chronic respiratory diseases could cost these countries roughly $ 7 trillion between 2011 and 2025 [3]. Correspondingly, estimates from India suggest that NCDs in India account for an economic burden in the range of 5–10% of GDP, significantly slowing down economic growth [4]. Since India is confronted by 'triple burden' of disease; in conjunction with infectious disease and injuries, burden posed by NCDs remains Achilles heel for underfunded health system. *Secondly,* NCDs have adverse impact on the financial stability of households with ailing members. National Health Accounts estimates divulge that abysmally low coverage of private health insurance coupled with dearth of government expenditure on prepayment mechanisms and public subsidies implied a reliance on out-of-pocket payments (58.7% of total health expenditure) for healthcare superfluously [5]. Consequently, households become vulnerable to catastrophic health expenditure and impoverishment due to health shocks which can culminate in the long term consequence of trans-generational cycle of poverty. The impact of NCDs on households is likely to be especially severe in LMICs where low-income populations, many of whom already experience extreme absolute poverty and precarious living conditions are especially vulnerable to impoverishment due to any degree of health spending. Thus, it is imperative to gauge an estimate of headcount of households susceptible to catastrophic health expenditure in order to bolster evidence backed policy decisions towards the trajectory of achieving Universal Health Coverage.

Traditionally, in India, healthcare financing has been mostly restricted to the supply side, focusing on strengthening of infrastructure and human resource. Albeit, the earmarked spending on NCDs and injuries by the government is less than 0.5% of GDP which is little more than one-fourth of total health spending of the country [6]. However, since 2007, myriad of publicly financed health insurance schemes lave been launched in India; both at the state level such as Rajiv Aarogyasri Health Insurance Scheme (RAS) in Andhra Pradesh, Rajiv Gandhi Jeevandayee Arogya Yojana (RGJAY) in Maharashtra, Chief Minister’s Comprehensive Health Insurance Scheme (CMCHIS) in Tamil Nadu and at center level such as Rashtriya Swasthya Bima Yojana (RSBY) and recently more expansive Pradhan Mantri Jan Arogya Yojana (PM-JAY) [7]. These demand-side financing mechanism entitle poor and vulnerable households to seek cashless secondary and tertiary hospitalization in select empaneled hospitals. However, no attempt has been made to gauge the extent of financial protection obtained for NCDs via these schemes. Studies have not been conducted specifically to discern catastrophic spending and impoverishment in NCD affected households. Thus, despite the policy relevance, there is a major lacunae in the evidence from population-based studies on the economic burden imposed by chronic non-communicable diseases in India.

India is also characterized by pervasive income inequalities in healthcare measures and in the absence of targeted and sustained interventions, the inequality gap is likely to extend. Even though, there is some limited evidence delving into the impact of socio-economic inequalities on incidence of catastrophic payments in Indian context [8] [9]; studies pertaining to NCDs have not been conducted. Analogously, there is substantial heterogeneity in the burden of NCDs and capacity to pay for healthcare between the states that needs to be examined. Hence, it is pertinent to conduct a disaggregated analysis at the granular level in order to generate the evidence for priority setting and discern how policies and implementation can be aligned to provide financial risk protection amongst the subjects of the state. Notwithstanding its implications, an enquiry into the regional variations are exiguous in literature. Further, the studies making an enquiry into the determinants of catastrophic payments are further scarce [10]; and most of the studies employed smaller sample size and were conducted at different time periods [11–14]; rendering inter-temporal and inter-regional comparisons arduous.

Given this backdrop and limitations in existing literature, we strived to undertake a succinct analysis of economic burden associated with NCDs at household level. Three-fold objectives are espoused in our study - *Firstly,* we carried out an assessment of the incidence and intensity of catastrophic health expenditures on NCDs at the national and disaggregated state level. *Secondly,* we attempted to quantify the extent to which catastrophic payments on NCDs results in impoverishment at the national and disaggregated state level and *Thirdly,* we unraveled the socio-economic determinants impacting the catastrophic health expenditures on NCDs in India. To our knowledge, this is the first study delving into the catastrophic payments exclusively related to NCDs in India. Our study has used most recent nationally representative large and robust dataset on morbidity, utilization and healthcare expenditures in India.

## Data and methods

### Data

Cross sectional individual level data was taken from two rounds of nationally representative National Sample Survey Organization surveys: Survey on Social Consumption (71st round) and Household Social Consumption in India: Health (75th round). These surveys were conducted under the stewardship of the Ministry of Statistics and Programme Implementation, Government of India and are representative at the state level as well. It collected information pertaining to households and individuals socio-economic background, morbidity status, utilization of healthcare services and healthcare expenditure on ambulatory, inpatient and delivery care. The survey rounds employed two-stage stratified design, with census villages and urban blocks as the first stage units (FSUs) for rural and urban areas respectively and households as the second stage units (SSUs). The sample size circumscribed 3, 35,499 and 5, 57,887 individuals (including death cases) encompassing 65,932 and 1, 13,823 households in 71st and 75th rounds respectively. The detailed information on survey design can be found in the afforementioned report [15, 16].

### Measures

Following outcome measures were gauged in the study: a) *Extent of out of pocket expenditure (OOPE) on healthcare* b) *Incidence and Intensity of catastrophic health expenditure as per income quintiles c) Impoverishing effects of catastrophic health expenditure d) Determinants influencing the catastrophic health expenditure*. Information on both medical and non-medical expenses was used from the survey to discern the extent of OOPE. Medical component incorporated information on doctor’s/surgeon’s fee, medicines, diagnostic tests, bed charges, physiotherapy, personal medical appliances and other consumables such as blood and oxygen etc. Whereas, non-medical components subsumed information on expenses incurred on transportation, food, lodging, and expenditure on escort and registration fees on account of treatment. OOPE is then defined as direct payments made by individuals to healthcare providers at the time of service use net of any reimbursements by medical insurance company or employer. Generally, catastrophic health expenditure is defined as OOPE for healthcare that exceeds a certain proportion of a household’s income with the consequence that households suffer the burden of disease [17]. However, varied definitions and thresholds are employed in the literature to calculate catastrophic health expenditure. Health expenditure is considered as catastrophic either - a) If a household’s financial contributions to the health system exceeds 40% of income remaining after subsistence needs have been met [18–20], in other words, defined as out of pocket payment for health care ≥40% capacity to pay or b) If a household’s financial contributions to the health system equals or exceeds 10% of total household expenditure [21–23]. There is also a gamut of studies that have taken varying levels of thresholds exhibiting sensitivity of different measures [24–26]. In this study, we computed the incidence of catastrophic expenditure using the 10% threshold of total expenditure and conducted the sensitivity analysis for varying thresholds at 5, 10, 25 and 40%.

The *Incidence* of catastrophic health expenditure was defined as the *headcount ratio* of the percentage of households whose OOP health payments exceed above-defined threshold in a given time period. The *Incidence* of catastrophic expenditure estimated by headcount however, doesn’t divulge information on how far (*Intensity*) the households spent beyond the threshold. This measure was estimated by *Overshoot* that computed the degree by which an average OOPE crossed the given threshold. Concomitantly, *Mean positive overshoot* measuring the degree by which the average OOPE by the households that have experienced the catastrophe exceeded the given threshold was assessed. In order to gauge the distribution of catastrophic health expenditure and Overshoot across income quintiles, *Concentration Indices* were computed.

The *Impoverishing* impact of healthcare spending delves into measurement of the extent of poverty due to OOP health spending incurred by the households. We used the methodology developed by Wagstaff and van Doorslaer [27] to estimate the impoverishing effect of OOPE. A measure of *Poverty Headcount* i.e. proportion of households that fell below poverty line was evaluated; a household was considered to be impoverished by OOP when its total per capita consumption spending fell below the poverty line defined by Planning Commission of India (Rangarajan Poverty Line). The measure was derived by calculating a) *Pre-payment headcount* and b) *Post-payment headcount. Pre-payment headcount* measured the percentage of individuals whose expenditure per adult equivalent was less than estimated poverty line for 2014 before spending for OOP health payments over entire reference population, while, *Post- payment* head count measured the percentage of individuals whose consumption per adult equivalent was less than estimated poverty line for 2014 after accounting for OOP health payments over the entire population. Hence, the difference in the headcounts before and after discounting OOP exhibited the impoverishment.

Although, *Poverty Headcount* captures the *Incidence* of Catastrophic expenditure, it precludes the evidence on the depth of poverty (*Intensity*), i.e. amount by which poor households fell short of reaching poverty line isn’t captured by this measure. Measure of *Poverty gap* however, encapsulates this information and was estimated to elucidate the amount by which out-of-pocket spending pushes the household below poverty line. The severity of the poverty was normalized by weighting the sum of the poverty gaps (as a proportion of poverty line) to uncover the *Normalized poverty gap.* However, in case of already poor household, the change in poverty gap is equal to the full amount of household’s health expenditure which was measured by *Mean Normalized poverty gap* reflecting the average depth of poverty among already poor due to OOPE.

In order to unravel the determinants driving the catastrophic expenditure; a gamut of household and health system’s level covariates were incorporated in the study guided by Andersen’s behavioral health model [28]. The choice of measures stemmed from previous literature, data availability from household survey and existence of routine availability of indicators through either routine management information systems or routine health surveys for scalability and generalizability. These measures are further classified and surmised as: a) *Predisposing* components incorporated into broad spectrum of factors encompassing i) Demographic characteristics such as age and gender of household head, age and gender mix of the household and household size and ii) Social-Structural characteristics such as educational status of household head, principal occupation of household and social group affiliation of the household. b) *Enabling characteristics* such as monthly per capita consumption expenditure quintiles, living condition index (Index obtained from composite score via principal component analysis upon amalgamation of indicators such as source of drinking water, access to latrine, waste disposal mechanism and primary source of energy for cooking), coverage of household by some pre-payment /insurance mechanism, source of financing for treatment of non-communicable diseases and choice of provider for treatment c) *Need based characteristics* such as NCD related hospitalization in the household, proportion of ailing members in household requiring NCD related care in the reference period and inter-state hospitalization and d) Contextual factors such as epidemiological transition level of state and spatial location of the household (rural/urban). Monthly per capita consumption expenditure was adjusted for household size and composition using the Oxford equivalence scale which was subsequently used as a proxy for economic status. The Oxford scale assigns the first adult in a household a weight of 1, each additional adult weight of 0.7, and each child (a person aged under 14) a weight of 0.5. The sum of these weights gives the number of adult equivalents in the household [29].

Data on both inpatient and ambulatory care was used in the analysis; since recall period for inpatient and ambulatory care expenditure is varied, it was converted and uniformed to the same recall period of one month in order to compute the OOPE and catastrophic expenditure. Accommodating for variability across two time periods, we adjusted the 2017–18 prices for inflation using Consumer Price Index time series data obtained from Handbook of Statistics on Indian Economy [30]. The list of NCDs in the survey data was mapped and extricated via ICD-10 classification.

### Statistical analysis

#### Estimates of incidence and intensity of catastrophic expenditures

The formula for share of out-of-pocket health expenditure is elucidated as follows:-

1 $$ {S}_i=\frac{H{E}_i}{T{E}_i} $$

Where, *HE*_*i*_ denotes *i* household’s out-of-pocket expenditure on healthcare consumption and *TE*_*i*_ denotes the household’s total consumption expenditure. From (1), consider *S*_*i*_ to be the share of healthcare expenditure for household *i* and Z as the threshold beyond which household *i* incurs catastrophic expenditure if, *S*_*i*_ > Z. The headcount is then, represented as:

2 $$ HC=\frac{1}{N}\sum \limits_{i=1}^N{D}_i $$

Where, N is the sample size and *D*_*i*_ is an indicator equal to 1 if *S*_*i*_ > Z and 0 otherwise.

Average overshoot measuring the degree by which average OOP expenditure exceeds the given threshold Z is depicted as:

3 $$ O=\frac{1}{N}\sum \limits_{i=1}^N{O}_i $$

Where, *O*_*i*_ is the amount by which household *i* share of health expenditure in total expenditure exceeds the threshold limit and is represented as:

4 $$ {O}_i={D}_i\left({S}_i-Z\right) $$

*HC* capturing the incidence of CHE occurring and *O* computing the intensity of CHE occurring are related through mean positive overshoot, which is defined as follows:

5 $$ MPO=\frac{O}{HC} $$

Thereby, implying *O* = *HC* × *MPO*, that can be interpreted as the catastrophic overshoot equals the fraction with catastrophic payments times the mean positive overshoot- the incidence times the intensity.

Further, inequality in incidence and intensity of catastrophic payments is computed by concentration index and is represented as:

6 $$ Concentration\ Index=\frac{2}{\mu }\  Cov\left(h,r\right) $$

Where, *μ* is the headcount ratio or overshoot and *Cov*(*h*, *r*) is the covariance of *HC* or *O* with relative rank of household based on total consumption expenditure. An index value of zero suggests absence of income/expenditure related inequalities, a positive value denotes concentration of the measure amongst the rich, whereas, negative value is indicative of concentration amongst the poor.

#### Estimates of poverty headcount and poverty gap

The equations below illustrate a parsimonious representation for examining the OOP payments on two basic measures of poverty- i) Headcount and ii) Poverty Gap.

The pre-payment *Poverty Headcount Ratio* is represented as:

7 $$ {H}^{pre}=\frac{1}{N}\kern0.5em \sum \limits_{i=1}^N{H}_i^{pre}=\sigma {p}^{pre} $$

Where, $\left({H}_i^{pre}\right)$ =1 if Monthly per capita expenditure (*TE*_*i*_) < Poverty Line *l* and 0 otherwise, *N* is the number of households in the sample and *σp*^*pre*^ is the proportion of population that is poor.

The average pre-payment *Poverty Gap Index* capturing amount necessary to raise an individual who is below poverty line up to that line is depicted as:

8 $$ {PG}^{pre}=\frac{1}{N}\ \sum \limits_{i=1}^N{pg}_i^{pre}=\sigma {g}^{pre} $$

Where, ${pg}_i^{pre}$ is the pre-payment poverty gap which is equal to (*l* − *TE*_*i*_) if (*TE*_*i*_) < Poverty Line *l* and 0 otherwise.

Normalized Poverty Gap which is the weighted sum of poverty gaps (as proportion of poverty line), gives more weight to observations that fall well below poverty line is computed as:

9 $$ {NPG}^{pre}=\frac{PG^{pre}}{l} $$

Further, normalized mean positive gap subsuming average depth of poverty amongst the poor is estimated as:

10 $$ {MNPG}^{pre}=\frac{\sum_{i=1}^N{pg}_i^{pre}}{\sum_{i=1}^N{H}_i^{pre}}=\frac{\sigma {g}^{pre}}{\upsigma {p}^{pre}} $$

which implies, *σg*^*pre*^= *σp*^*pre*^× *MNPG*^*pre*.^

Poverty indices for post-payment expenditure are obtained by subtracting household health expenditure on inpatient and outpatient care *HE*_*i*_ from pre- payment expenditure (*TE*_*i*_− _*HEi*_). The impoverishment impact of OOPE is then, estimated by deducting pre-payment indices from post- payment indices which are surmised below:-

11 $$ Headcount:{P}^{HC}=\kern0.5em {H}^{post}-{H}^{pre} $$

12 $$ Poverty\  Gap:{P}^G={PG}^{post}-{PG}^{pre} $$

13 $$ Normalized\ Poverty\  Gap:{P}^{NPG}={NPG}^{post}-{NPG}^{pre} $$

14 $$ Mean\ Normalized\ Poverty\  Gap:{P}^{MNPG}={MNPG}^{post}-{MNPG}^{pre} $$

#### Determinants of catastrophic health expenditure

Determinants of Catastrophic Health Expenditure were determined by multivariate logistic regression model:

15 $$ {S}_i=\mathit{\ln}\left(\frac{\hat{y}}{1-\hat{y}}\right)={\beta}_0+{\beta}_1{X}_1+{\beta}_2{X}_2+\dots \dots \dots +{\beta}_n{X}_n $$

Where, dependent variable following the definition of Catastrophic Health Expenditure is dichotomous i.e. *S*_*i*_ takes the value of 1, if a household’s healthcare expenditure (*HE*_*i*_) exceeds the 10% threshold of total household expenditure (*TE*_*i*_) and 0 otherwise and *X*_1_…. . *X*_*n*_ are the legion of covariates subsuming socio-economic and demographic characteristics of the households.

Statistical analysis of data was conducted with STATA 13 statistical software package and weighted estimates were considered accounting for complex multistage sampling design of survey rounds. Further, the maps depicting inter-state heterogeneities in incidence of catastrophic payments and Impoverishment impact were generated using ArcGIS (ArcMap 10.7).

## Results

### Pattern of non-communicable disease burden in India

The pattern of major NCDs across rural and urban sectors in India is depicted in Table 1. Cardiovascular diseases was reported to be the leading cause of NCD burden in India which also witnessed rise in incidence over the period of time. Amongst the individuals treated for NCDs in rural areas in 2014, 17.7% were treated for cardiovascular diseases which further increased to 20.7% in 2017–18. However, the burden was more pronounced for urban areas as more than one-fourth of the total NCD burden was attributed to cardiovascular diseases. A major divergence between the regions was exhibited for diabetes, 9.8% of total NCD burden in 2014 in rural areas was associated with diabetes which was extended to 14.9% in 2017–18, whereas the burden was twice of that in urban areas constituting 20.1 and 23.2% of total burden. The musculoskeletal diseases were third major cause of NCDs in India having more incidence in rural areas (19.9% in 2014 and 15.8% in 2017–18) vis-à-visurban areas (12.9% in 2014 and 11.9% in 2017–18). Further, neurological and psychiatric disorders contributed significantly to the NCD burden, conversely, the burden of cancer was only 1% for both the regions in 2014 which marginally increased in 2017–18. Other NCDs, encompassing conditions such as  genitourinary, eye, ear, chronic respiratory and endocrine related ailments were also major contributors of the burden, however, it declined over the years from 35 and 27.8% in rural and urban areas respectively in 2014 to 29.7 and 22.7% in rural and urban areas respectively in 2017–18.

**Table 1:** Pattern of Major Non-Communicable Diseases Across Rural and Urban Sectors (in %)

	Rural		Urban	
	2014	2017–18	2014	2017–18
**Cancer**	1	1.4	1	1.3
**Cardiovascular**	17.7	20.7	25.1	27.8
**Diabetes**	9.8	14.9	20.1	23.2
**Respiratory**	6.8	9.3	5.1	6.7
**Musculoskeletal**	19.9	15.8	12.9	11.9
**Neurological**	9.8	8.2	8	6.4
**Other NCDs**	35	29.7	27.8	22.7

A sharp increase in comorbidity was observed from 2014 to 2017–18 in both rural and urban India. Proportion of individuals having two distinct NCD conditions (comormidity) rose from 1.5% in 2014 to 7.98% in 2017–18 in rural regions and exhibited an increase form 2.05% in 2014 to 10.94% in 2017–18 in urban regions. The presence of multimorbidity with three distinct NCD conditions also increased over the years as tabulated in Table 2, thereby, insinuating greater burden and costs.

**Table 2:** Presence of NCD related comorbidity Across Rural and Urban sectors (in %)

	Rural		Urban	
	2014	2017–18	2014	2017–18
**No Comorbidity**	98.28	89.06	97.71	85.08
**Comorbidity(2 NCDs)**	1.5	7.98	2.05	10.94
**Comorbidity (3 NCDs)**	0.2	2.35	0.21	3.4
**Comorbidity (< than 3NCDs)**	0.02	0.61	0.03	0.58

### Socio-economic and Demograhic profile of study population

Table 3 captures the summary statistics of variables incorporated in study for years 2014 and 2017–18. Majority of dwellings had 4–6 members habitating in household units in both 2014 (53.2%) and 2017–18 (52.8%). The structure and dynamics of occupational categories in both the study years was consonant with each other , where majority of households were primarily self-employed (46.6 and 45.7% in 2014 and 2017–18 respectively). However, less than quarter households i.e. 20.6% in 2014 and 20.9% in 2017–18 were employed as regular wage/salaried workers. Rural-Urban mix was also cognate in both study years with 38% households residing in urban areas. Indian society is socially stratified into various hierarchical groups where Scheduled Caste/Scheduled Tribes (SC/ST) and Other Backward Castes (OBC) constitute the marginalized groups. In 2014; 22.7 and 44.7% households were belonging to SC/ST and OBC groups; whereas the same distribution was 22.9% for SC/ST and 41.8% for OBC’s respectively in 2017–18. Only one-seventh households in 2014 (14.1%) and 2017–18(14.1%) were female headed households and majority of household heads were aged less than 60 years in both 2014 (68.8%) and 2017–18 (67.3%). Another enabling factor of educational status of household head also varied between the households. A colossal 42.6 and 38.7% hosuehold heads were illiterate/dropped out at primary level in 2014 and 2017–18 resepctively;  conversely, an exiguous 9.4% household heads in 2014 and 11.2% in 2017–18 were graduate and above. More than 80% household heads were married on the day of survey and more than one-third (38.3% in 2014 and 40.3% in 2017–18) were suffering from some chronic ailment. Financial risk protection was meagre as only one-fourth households (25.6% in 2014 and 26.9% in 2017–18) had insurance coverage, with rest of the households being vulnerable to health shocks. Health expenditures are contingent upon the age composition of household members as relatively more expenditure is incurred upon elderly and children. Larger proportion of households (39.9%) in 2014 and 40.3% in 2017–18 had no children/elderly. Treatment seeking behaviour predominantly was characterized by visits to private providers as 66.7% households in 2014 and 63% in 2017–18 sought treatment from private providers for various spells of treatments/hospitalization and majority of households financed the treatment costs via household income/ savings (89.4% in 2014 and 91.4% in 2017–18). More than one third households(38.5% in 2014 and 45.6% in 2017–18) were characterized by poor living conditions in terms of access to clean cooking fuel, drinking water, latrine and drainage which were represented as high risk factor households. Greater proportion of households were residing in states at higher middle/high level of ETL(64.7% in 2014 and 65.8% in 2017–18) having greater burden of NCDs and most households (98.2% in 2014 and 98.4% in 2017–18) sought care within the  administrative boundaries of their state for NCD- related hospitalization treatment.

**Table 3:** Descriptive Statistics of households

VARIABLES		2014 (***N*** = 26,816)	2017–18 (***N*** = 38,835)
		% (95% CI)	% (95% CI)
**Age of household head**	Less than 60	68.83 [67.37,70.25]	67.31 [65.99,68.59]
	60 or more	31.17 [29.75,32.63]	32.69 [31.41,34.01]
**Gender of household head**	Male	85.92 [84.78,86.98]	85.88 [84.88,86.82]
	Female	14.08 [13.02,15.22]	14.12 [13.18,15.12]
**Age composition of household members**	No children or old	39.86 [38.33,41.42]	42.41 [41.01,43.81]
	With children but no older people	14.18 [13.19,15.22]	11.72 [10.91,12.59]
	With older people but no children	28.28 [26.86,29.73]	30.15 [28.86,31.46]
	With both children and older people	11.61 [10.74,12.53]	9.18 [8.51,9.91]
	Older people only	6.08 [5.33,6.93]	6.54 [5.89,7.262]
**Household gender composition**	All female	4.24 [3.6,5]	4.768 [4.18,5.44]
	Both male and female	94.61 [93.8,95.33]	93.71 [92.96,94.38]
	All male	1.14 [0.85,1.51]	1.52 [1.23,1.893]
**Household size**	1–3 member	29.59 [28.14,31.09]	31.99 [30.67,33.34]
	4–6 member	53.25 [51.69,54.81]	52.83 [51.44,54.22]
	7 or above members	17.15 [16.05,18.31]	15.18 [14.25,16.16]
**Educational Status of household head**	Illiterate or below primary	42.64 [41.03,44.27]	38.77 [37.34,40.21]
	Primary completed	12.9 [11.93,13.95]	13.56 [12.63,14.53]
	Middle completed	15.01 [13.93,16.15]	14.26 [13.31,15.26]
	Secondary/Senior secondary	20.06 [18.82,21.36]	22.17 [21.07,23.32]
	Graduation or above	9.39 [8.5,10.36]	11.25 [10.36,12.2]
**Principal Occupation of Household**	Regular wage/salary	20.65 [19.43,21.92]	20.92 [19.81,22.07]
	Self-employed	46.58 [45.01,48.17]	45.7 [44.29,47.13]
	Casual Labour	22.33 [21,23.17]	22.51 [21.28,23.79]
	Others	10.44 [9.46,11.52]	10.87 [10.05,11.75]
**Social Group**	Scheduled Caste/ Tribe	22.71 [21.32,24.16]	22.93 [21.69,24.23]
	Other Backward Castes	44.73 [42.99,46.49]	41.84 [40.35,43.34]
	Others	32.56 [30.97,34.19]	35.23 [33.8,36.69]
**Monthly Per Capita Expenditure Quintiles**	Poorest	21.38 [20,22.84]	22.26 [20.99,23.58]
	Poor	19.33 [18.08,20.65]	19.89 [18.74,21.08]
	Middle	19.7 [18.49,20.98]	19.64 [18.55,20.78]
	Rich	19.18 [18.04,20.38]	19.27 [18.21,20.37]
	Richest	20.4 [19.14,21.72]	18.95 [17.86,20.1]
**Living Condition Index**	Lowest	38.49 [36.81,40.2]	38.24 [36.75,39.74]
	Middle	19.25 [17.95,20.61]	13.5 [12.54,14.52]
	Highest	42.26 [40.58,43.96]	48.26 [46.75,49.78]
**Whether covered by Insurance**	No	74.39 [72.79,75.94]	73.11 [71.65,74.53]
	Yes	25.61 [24.06,27.21]	26.89 [25.47,28.35]
**Finance source**	Household income/savings	89.4 [88.57,90.17]	91.37 [90.65,92.03]
	Borrowings	7.54 [6.9,8.22]	4.37 [3.913,4.878]
	Other sources	3.06 [2.62,3.6]	4.27 [3.78,4.81]
**Care seeking for NCD related treatment**	Only Public	22.43 [21.14,23.78]	27.61 [26.35,28.91]
	Both Public and Private	10.89 [10.16,11.66]	9.39 [8.82,9.99]
	Only Private	66.68 [65.21,68.12]	63 [61.64,64.34]
**Hospitalization for NCD in household**	No	75.79[75.03,76.54]	82.16[81.62,82.68]
	Yes	24.21[23.46,24.97]	17.84[17.32,18.38]
**Household members requiring care**	One	83.11[82,84.16]	84.16[82.85,81.90]
	Two	12.78[11.88,13.73]	13.73[13.04,12.25]
	Three or More	4.12[3.64,4.66]	4.66[4.11,3.73]
**Inter-state hospitalization**	No	98.2 [97.96,98.42]	98.4 [98.21,98.57]
	Yes	1.8 [1.58,2.04]	1.6 [1.43,1.79]
**Epidemiological transition level of state**	Low-ETL	30.09 [28.33,31.92]	29.27 [27.74,30.84]
	Lower-middle ETL	5.2 [4.5,6]	4.88 [4.21,5.65]
	Higher-middle ETL	39.86 [37.9,41.86]	45.63 [43.9,47.38]
	High ETL	24.84 [23.09,26.68]	20.22 [18.98,21.53]
**Location**	Rural	61.92 [60.31,63.5]	62.01 [60.59,63.41]
	Urban	38.08 [36.5,39.7]	37.99 [36.59,39.41]

### Incidence and intensity of catastrophic health spending on NCDs in India

Table 4 summarizes the incidence (headcount) of CHE incurred on account of treatment of NCDs in India. The results suggests that in 2014, overall CHE ranged from 32.4 to 79.8% across alternative threshold levels of share of pre-payment expenditure, allowing to explore the sensitivity of results. An inverse relationship was observed between catastrophic incidence and various thresholds. The estimates in 2017–18 declined marginally to the range of 27.2–77.1% as the threshold is decreased from 40 to 10%. In 2014, at 10% threshold, the incidence of catastrophic payments for poorest quintile was 7% more than for richest quintile; which further dilated to 20% in 2017–18. On an average, incidence of catastrophic payments, at 10% threshold exhibited a slump from 68.1% in 2014 to 63.6% in 2017–18. Statistically significant negative value of concentration index for both the study years indicated concentration of catastrophic payments towards the poor. The inequality in the incidence of catastrophic expenditures disfavoring poor augmented between 2014 (CI = − 0.008 to − 0.106) and 2017–18 (CI = − 0.035 to − 0.175). The estimates also approached higher values as threshold was increased from 5 to 40%.

**Table 4:** Results for Incidence of NCD-related Catastrophic Health Expenditure Across the years in India

Headcount	Parameters	(2014)				(2017–2018)			
		5%	10%	25%	40%	5%	10%	25%	40%
**Poorest**	**%**	79.51	70.19	53.47	41.04	84.00	72.9	52.27	38.64
	**S.E**	0.016	0.018	0.019	0.018	0.013	0.015	0.017	0.016
	**CI**	[76.40–82.62]	[66.68–73.7]	[49.8–57.13]	[37.45–44.62]	[81.51–86.49]	[69.91–75.90]	[48.97–55.56]	[35.44–41.84]
**Poorer**	**%**	81.11	72.41	50.98	37.61	79.12	68.57	44.1	32.07
	**S.E**	0.015	0.016	0.019	0.019	0.014	0.016	0.016	0.015
	**CI**	[78.24–83.99]	[69.21–75.61]	[47.3–54.67]	[33.96–41.26]	[76.35–81.88]	[65.49–71.65]	[40.90–47.31]	[29.13–35.02]
**Middle**	**%**	79.99	67.73	43.76	28.66	76.77	61.59	37.61	25.14
	**S.E**	0.016	0.018	0.018	0.015	0.013	0.016	0.015	0.013
	**CI**	[76.89–83.09]	[64.22–71.23]	[40.33–47.2]	[25.65–31.67]	[74.14–79.4]	[58.5–64.7]	[34.68–40.55]	[22.61–27.66]
**Richer**	**%**	80.65	67.12	41.3	27.12	73.95	60.3	35.19	21.94
	**S.E**	0.014	0.016	0.016	0.014	0.014	0.016	0.015	0.012
	**CI**	[77.89–83.41]	[63.93–70.3]	[38.12–44.47]	[24.37–29.88]	[71.30–76.61]	[57.24–63.35]	[32.34–38.04]	[19.62–24.26]
**Richest**	**%**	77.83	63.31	38.57	26.76	70.51	53	26.68	16.09
	**S.E**	0.015	0.016	0.016	0.015	0.014	0.015	0.013	0.010
	**CI**	[74.96–80.71]	[60.1–66.54]	[35.36–41.79]	[23.89–29.63]	[67.73–73.3]	[50.04–55.93]	[24.20–29.16]	[14.04–18.13]
**Total**	**%**	79.79	68.14	45.7	32.36	77.12	63.61	39.62	27.19
	**S.E**	0.007	0.008	0.008	0.008	0.006	0.007	0.007	0.006
	**CI**	[78.44–81.14]	[66.61–69.67]	[44.12–47.28]	[30.87–33.84]	[75.89–78.34]	[62.20–65.03]	[38.22–41.02]	[25.95–28.45]
**Concentration index (Headcount)**	**Index value**	−0.008	−0.027	−0.077	−0.106	−0.035	−0.065	−0.131	−0.175
	**S.E**	0.005	0.007	0.010	0.013	0.005	0.006	0.010	0.013
	***P*-value**	0.10	0.00	0.00	0.00	0.00	0.00	0.00	0.00

Table 5 captures catastrophic overshoot and mean positive overshoot, defined as mean level by which out-of-pocket expenditure on illness of  a household reporting the catastrophic health expenditure exceed the household expenditure. During 2014, the intensity of catastrophe (i.e. the overshoot) at 10% threshold was 42.76% i.e. on an average, households spent a colossal 42.76% beyond the 10% catastrophic threshold. Albeit, CHE is not experienced by all the households; estimates of mean positive overshoot demonstrated that, on an average, OOP health payments for households with NCDs incurring catastrophic expenditure, spent 62% higher than the 10% threshold of total consumption. Thus, these households spent 72% (sum of mean positive overshoot and threshold) of their total expenditure on the treatment of NCD. The sensitivity analysis demonstrated that estimates of overshoot dropped as the threshold was raised from 5 to 40% while reverse trend was exhibited in mean positive overshoot. The average OOP paid as a share of total expenditure declined over the years. The intensity of payments plummeted in 2017–18, with overshoot of 34.9% and mean positive overshoot of 54.8% at 10% threshold. However, poorest households endured greatest overshoot and richest suffered the least, connoting the unequal distribution. The concentration index was − 0.18 in 2014 disfavoring the poor which further widened to − 0.23 in 2017–18; thereby deepening inequities against the poor over the period of years.

**Table 5:** Results for Intensity of NCD-related Catastrophic Health Expenditure Across the years in India

Headcount	Parameters	(2014)				(2017–2018)			
		5%	10%	25%	40%	5%	10%	25%	40%
**Poorest**	**%**	69.1	65.39	56.2	49.22	61.38	57.48	48.23	41.55
	**S.E**	0.063	0.063	0.062	0.061	0.039	0.039	0.038	0.037
	**CI**	[56.72–81.48]	[53.05–77.72]	[44.03–68.37]	[37.24–61.20]	[53.68–69.09]	[49.81–65.15]	[40.75–55.73]	[34.29–48.81]
**Poorer**	**%**	54.53	50.69	41.56	34.99	44.72	41.02	32.89	27.24
	**S.E**	0.054	0.054	0.053	0.052	0.036	0.036	0.035	0.034
	**CI**	[43.91–65.16]	[40.11–61.28]	[31.17–51.95]	[24.84–45.15]	[37.7–51.73]	[34.03–44.01]	[26.04–39.73]	[20.55–33.94]
**Middle**	**%**	38.05	34.36	26	20.73	32.28	28.83	21.57	16.91
	**S.E**	0.022	0.022	0.021	0.019	0.015	0.015	0.014	0.013
	**CI**	[33.75–42.35]	[30.11–38.61]	[21.96–30.05]	[16.91–24.54]	[29.3–35.27]	[25.90–31.77]	[18.86–24.29]	[14.42–19.41]
**Richer**	**%**	34.48	30.79	22.84	17.74	28.78	25.44	18.53	14.39
	**S.E**	0.017	0.017	0.016	0.015	0.018	0.018	0.017	0.016
	**CI**	[31.09–37.86]	[27.46–34.13]	[19.70–25.98]	[14.81–20.68]	[25.28–32.29]	[21.97–28.90]	[15.24–21.81]	[11.27–17.51]
**Richest**	**%**	34.41	30.89	23.48	18.69	21.03	17.97	12.33	9.2
	**S.E**	0.020	0.019	0.018	0.017	0.010	0.010	0.008	0.008
	**CI**	[30.56–38.26]	[27.09–34.68]	[19.91–27.05]	[15.35–22.02]	[19.12–22.95]	[16.10–19.83]	[10.66–13.99]	[7.72–10.67]
**Total overshoot**	**%**	46.45	42.76	34.35	28.6	38.43	34.92	27.42	22.5
	**S.E**	0.019	0.019	0.018	0.018	0.013	0.013	0.012	0.012
	**CI**	[42.77–50.13]	[39.1–46.42]	[30.76–37.93]	[25.1–32.1]	[35.94–40.91]	[32.45–37.39]	[25.02–29.81]	[20.2–24.81]
**Mean positive overshoot (MPO)**	**%**	58.21	62.75	75.16	88.36	49.83	54.89	69.19	82.74
	**S.E**	0.02	0.03	0.04	0.05	0.01598	0.02	0.03	0.04
	**CI**	[53.73–62.69]	[57.6–67.9]	[67.86–82.5]	[78.55–98.18]	[46.7–52.96]	[51.2–58.58]	[63.67–74.70]	[75.19–90.29]
**Concentration index (Overshoot)**	**Index value**	−0.168	−0.182	−0.213	−0.238	−0.217	−0.234	−0.272	−0.298
	**S.E**	0.027	0.030	0.036	0.043	0.020	0.022	0.027	0.032
	***P*-value**	0.00	0.00	0.00	0.00	0.00	0.00	0.00	0.00

### Impoverishing and poverty gap due to OOP in India

Poverty levels obtained using post-payment income (after making OOP healthcare payments) were higher than those obtained using pre-payment incomes. As represented in Table 6, in 2014, 8.5% households not counted as living in extreme poverty would be considered poor if spending on healthcare is discounted from household resources. This represents a substantial upsurge of 10.8% in the poverty estimates. Poverty gap on account of OOPE also rose to the extent of 76.37% amounting to INR 318.45. Expressed as a percentage of the poverty line, the poverty gap increased by 75.3% when health payments are netted out of household consumption. Mean positive poverty, capturing severity of impoverishment amongst the poor, increased by 27.7%, insinuating the deepening of poverty amongst already poor. Relatively, this translates to 58.2% deepening of poverty of already poor on account of NCD related expenses. The proportion of Indians who were pushed under Below Poverty Line attributable to the expenses on NCD- related treatment increased furthermore to 12.43% in 2017–18. Also, normalized poverty gap and normalized mean poverty gap extended by 38.53 and 20.52% vis-à-vis 2014 denoting further deepening of poverty post- payment of health expenses.

**Table 6:** Impoverishing effects of Catastrophic Health Expenditures due to NCDs in India

Year	2014		2017–18	
	Absolute	Relative	Absolute	Relative
**Poverty headcount**	8.50%	10.80%	12.43%	19.62%
	0.004		0.004	
**Poverty Gap (in INR 2014 prices)**	318.45	76.37%	307.99	115.87%
	11.55		10.5	
**Normalized poverty gap**	28.23%	75.39%	27.57%	113.92%
	0.011		0.010	
**Normalized mean poverty gap**	27.73%	58.19%	30.11%	78.71%

### Inter-state heterogeneities in catastrophic and impoverishment headcount

**Fig. 1:**
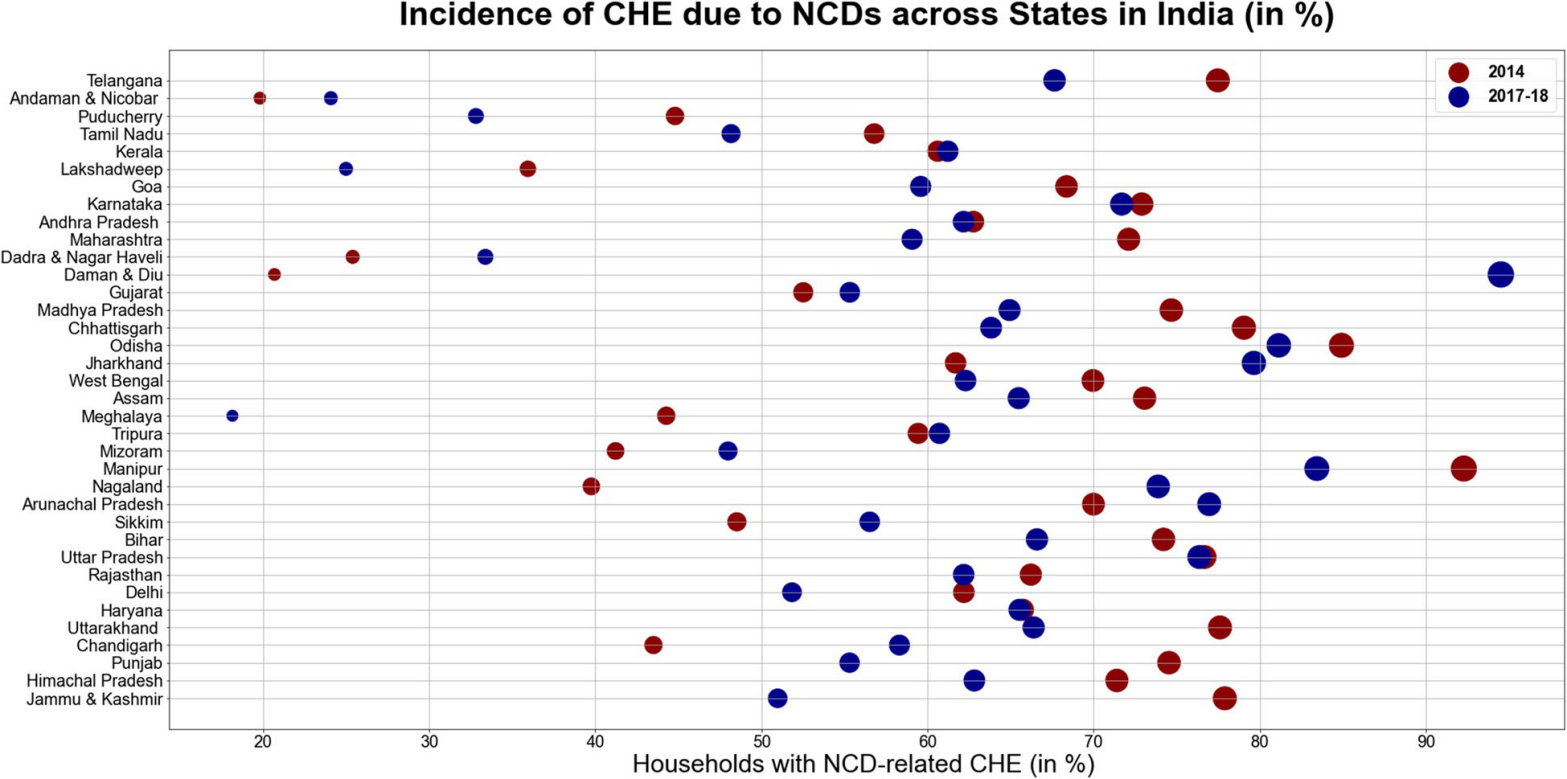
Incidence of Catastrophic Health Expenditure due to NCDs Across States in India(%)

**Fig. 2:**
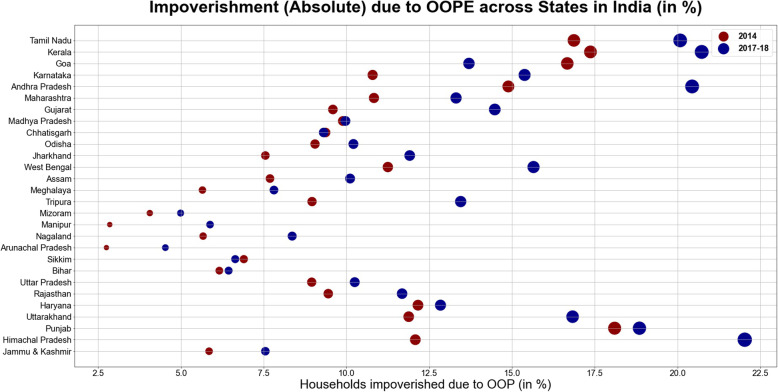
Impoverishment(Absolute) Due to Catastrophic Payments Across States in India (in %)

Table 7 and Fig. 1 represents inter-state heterogeneities in the extent of catastrophic health expenditures in India for the period from 2014 to 2017–18. Overall, all the states exhibited enormous burden of catastrophic expenditure amongst the households with member(s) ailing from NCDs. In 2014, the incidence was higher amongst the major states of India such as Karnataka (72.9%), Madhya Pradesh (74.68%), Odisha (79.05%), Uttar Pradesh (76.68%), Bihar (74.21%), Assam (73.07%), Chhattisgarh (79.05%), Telangana (77.48%) and other hill states such as Jammu and Kashmir (77.90), Himachal Pradesh (71.40%) and Uttarakhand (77.61%). However, the incidence was relatively low in the Union Territories such as Andaman and Nicobar Islands (19.80%), Daman & Diu (20.68%), Dadra and Nagar Haveli (25.39%), Lakshadweep (35.94%), Chandigarh (43.50%) and Puducherry (44.8%). The change from 2014 to 2017–18 divulged a mixed trend, with large and hilly states such as Jammu and Kashmir (− 26.9%), Madhya Pradesh (− 9.74%), Punjab (− 19.2%), Chhattisgarh (− 15.2), Maharashtra (− 13.0%), Uttarakhand (− 11.2%), Telangana (− 9.83%), Tamil Nadu(− 8.62%), West Bengal(− 7.67%), Bihar(− 7.62%), Assam(− 7.58%) witnessing a decline in catastrophic expenditure; while, smaller Northeastern states and Union Territories such as Nagaland(34.1%), Daman and Diu(73.8%), Dadra and Nagar Haveli(7.9%), Sikkim(8.0%), Arunachal Pradesh(6.9%) and Mizoram(6.7%) marking a rise in catastrophic payments. The detailed state-wise estimates of monthly per capita consumption expenditure, out- of- pocket payments and level of impoverishment, are provided in Table 1, Additional file 1: Appendix. The eight socio-economically backward empowered action group (EAG) states comprising of Bihar, Chhattisgarh, Jharkhand, Madhya Pradesh, Odisha, Rajasthan and Uttarakhand experienced higher burden of CHE on an average (74.4% in 2014 and 70.1% in 2017–18 respectively) vis-à-vis non EAG states and union territories (56.67% in 2014 and 56.23% in 2017–18 respectively). Further, the incidence of catastrophic payments as illustrated in Table 7, Fig. 2 and Table 5; (Additional file 1: Appendix), was found to be relatively high either in the states which are at the lowest (70.5 and 64.3% in 2014 and 2017–18, on an average) or  the highest stage(66.3 and 57.4% on an average in 2014 and 2017–18 respectively) of the Epidemiological transition level (ETL which is defined on the basis of ratio of Disability-Adjusted Life Years (DALYs), computed as the sum of years of potential life lost due to premature mortality and the years of productive life lost due to disability from communicable disease to those from non-communicable disease and injuries combined).

**Table 7:** State-Wise Estimates for Incidence of Catastrophic Health Expenditure amongst Households with NCD burden

State	Households incurring Catastrophic health expenditure (%) in 2014		Households incurring Catastrophic health expenditure (%) in 2017–18	
	%	CI	%	CI
Jammu & Kashmir	77.9	[66.97–85.97]	50.98	[43.42–58.5]
Himachal Pradesh	71.4	[60.2–80.48]	62.82	[54.43–70.49]
Punjab	74.54	[66.01–81.53]	55.31	[48.48–61.94]
Chandigarh	43.5	[15.9–75.82]	58.31	[37.82–76.28]
Uttarakhand	77.61	[59.34–89.17]	66.39	[51.5–78.61]
Haryana	65.74	[54.36–75.56]	65.54	[55.12–74.64]
Delhi	62.19	[46.7–75.53]	51.84	[36.48–66.85]
Rajasthan	66.22	[59.86–72.04]	62.18	[56.08–67.91]
Uttar Pradesh	76.68	[72.5–80.4]	76.36	[72.48–79.85]
Bihar	74.21	[64.67–81.9]	66.59	[55.37–76.2]
Sikkim	48.52	[26.72–70.9]	56.52	[40.71–71.11]
Arunachal Pradesh	69.99	[50.82–84.03]	76.96	[67.53–84.29]
Nagaland	39.76	[20.02–63.52]	73.89	[59.35–84.57]
Manipur	92.29	[86.7–95.65]	83.44	[76.95–88.38]
Mizoram	41.22	[25.41–59.07]	47.99	[35.51–60.72]
Tripura	59.44	[46.74–70.99]	60.72	[52.24–68.59]
Meghalaya	44.27	[22.72–68.21]	18.15	[12–26.51]
Assam	73.07	[61.71–82.04]	65.49	[55.52–74.27]
West Bengal	69.96	[65.43–74.12]	62.29	[58.22–66.2]
Jharkhand	61.69	[48.41–73.43]	79.65	[71.41–85.99]
Odisha	84.92	[79.64–89.03]	81.15	[75.85–85.51]
Chhattisgarh	79.05	[65.48–88.25]	63.83	[52.35–73.92]
Madhya Pradesh	74.68	[67.67–80.61]	64.94	[54.75–73.93]
Gujarat	52.52	[46.22–58.75]	55.32	[47.87–62.54]
Daman & Diu	20.68	[3.72–63.72]	94.52	[64.03–99.41]
Dadra & Nagar Haveli	25.39	[12.36–45.07]	33.38	[20.88–48.75]
Maharashtra	72.11	[66.98–76.72]	59.07	[54.57–63.42]
Andhra Pradesh (Undivided)	66.54	[61.2–71.48]	63.31	[58.58–67.8]
Karnataka	72.9	[65.96–78.88]	71.69	[64.92–77.6]
Goa	68.37	[52.43–80.92]	59.59	[41.73–75.22]
Lakshadweep	35.94	[17.47–59.79]	24.99	[16.46–36.05]
Kerala	60.62	[56.34–64.75]	61.22	[57.82–64.51]
Tamil Nadu	56.79	[51.21–62.2]	48.17	[43.02–53.35]
Puducherry	44.8	[30.5–60.02]	32.81	[17.67–52.64]
Andaman & Nicobar Islands	19.8	[8.86–38.53]	24.08	[14.88–36.54]

**Fig. 3:**
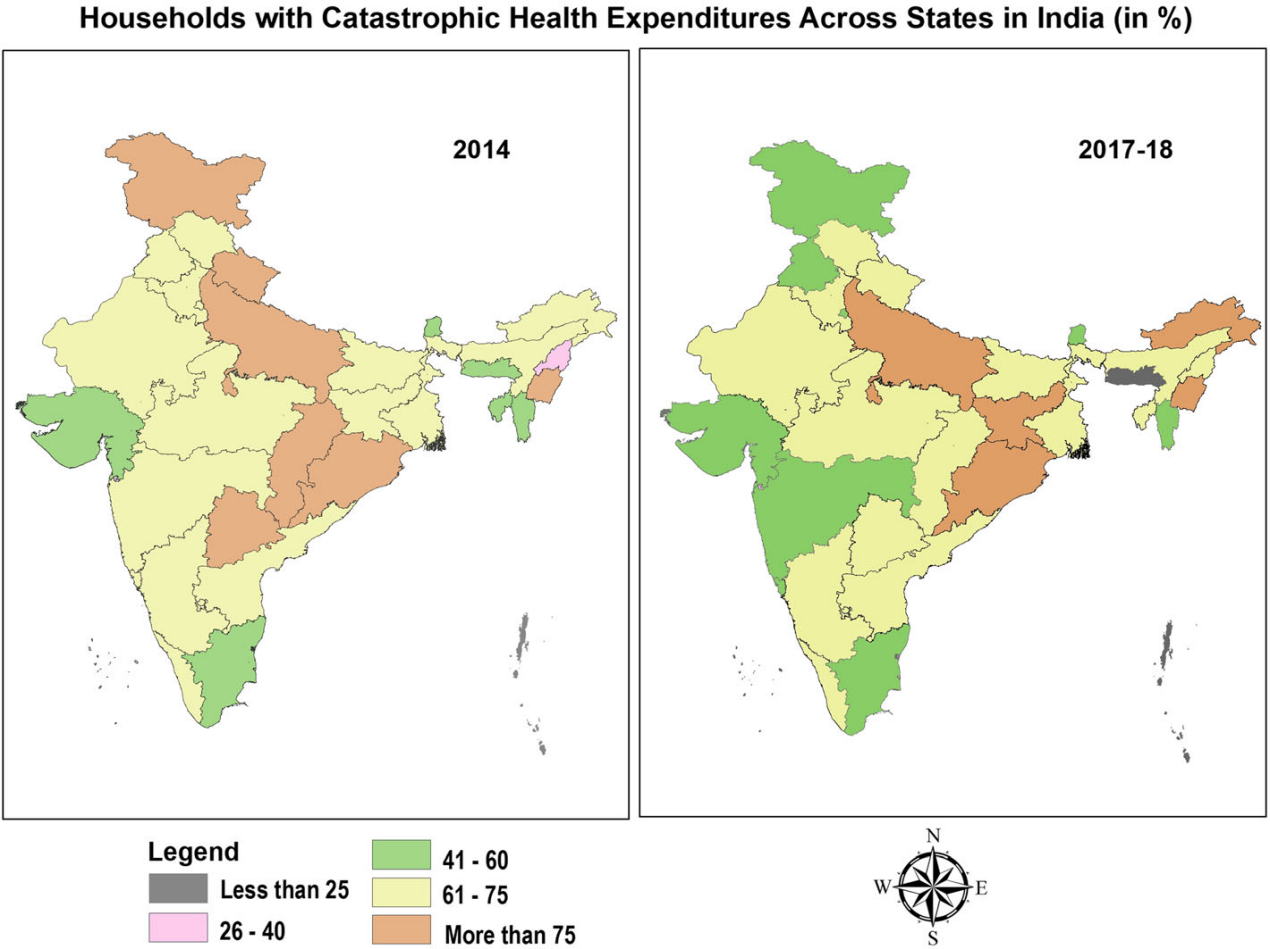
Households with Catastrophic Health Expenditures Across States in India (in %)

**Fig. 4:**
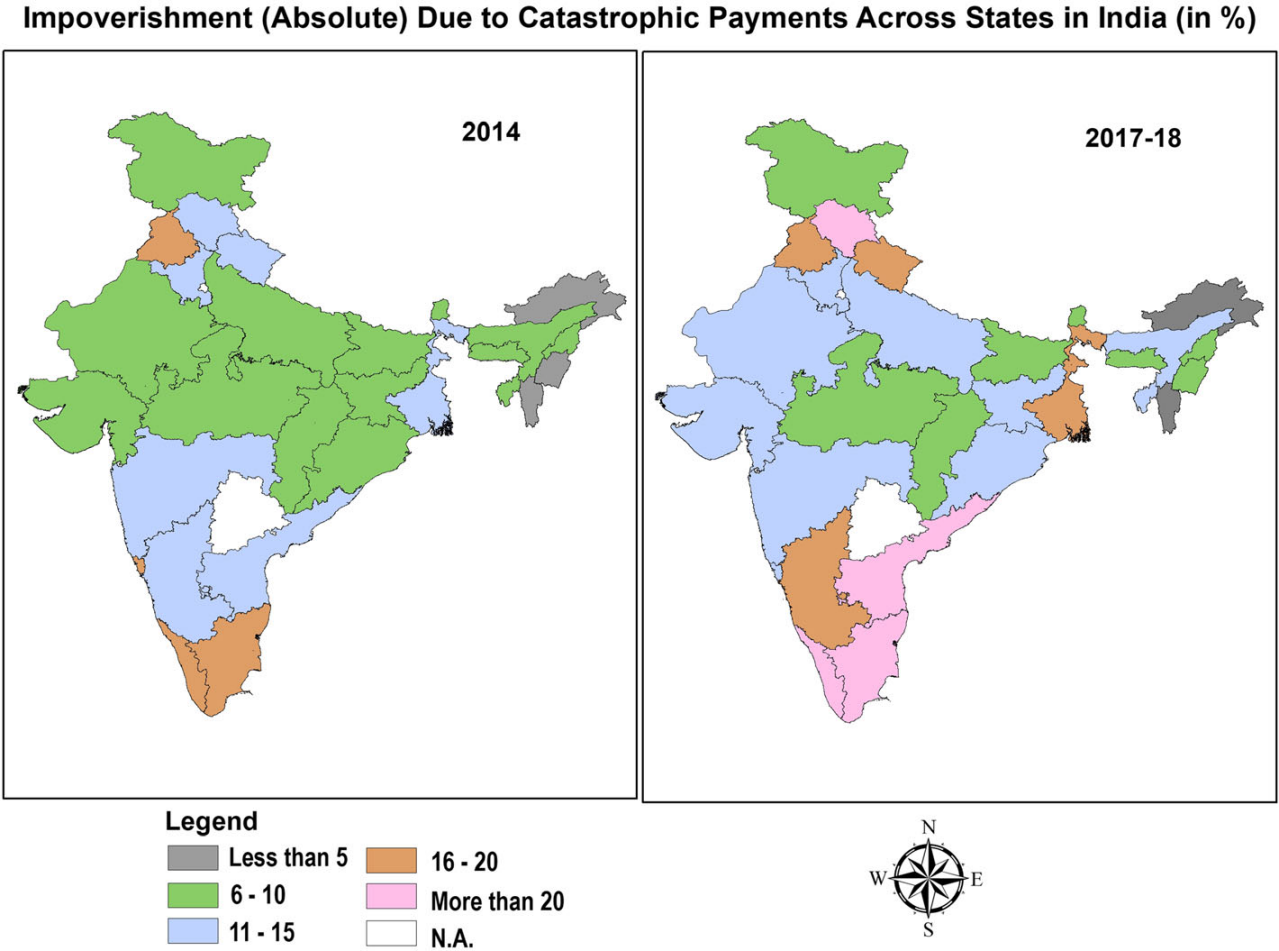
Impoverishment (Absolute) Due to Catastrophic Payments Across States in India (in %)

Significant inter-state variation with marked disparities between best performing and worst performing states was found in the estimates of impoverishment (Table 8). Amongst the states with highest burden of NCDs, absolute impoverishment for households on account of treatment of NCDs was highest in Kerala (17.4% in 2014 and 20.7% in 2017–18), Tamil-Nadu (16.9% in 2014 and 20% in 2017–18), Goa (16.7% in 2014 and 13.7% in 2017–18), Himachal Pradesh (12.1% in 2014 and 22.0% in 2017–18) and Punjab (18.1% in 2014 and 18.8% in 2017–18). All these states belonged to the highest Epidemiological Transition Level (ETL) group characterized with burden of disease which is skewed disproportionately towards the NCDs such as cardiovascular disease, diabetes, respiratory problems and cancer etc. [1]. Moreover, other states like West Bengal (11.25% in 2014 and 15.6% in 2017–18) and Andhra Pradesh (14.9% in 2014 and 20.4% in 2017–18) which are at the higher-middle group of ETL also experienced higher incidence of poverty. Conversely, states which are at the low or lower- middle group such as Bihar (6.2% in 2014 and 6.4% in 2017–18), Meghalaya (5.6% in 2014 and 7.8% in 2017–18), Arunachal Pradesh (2.7% in 2014 and 4.5% in 2017–18) and other hilly North-Eastern states observed relatively less extent of impoverishment emanating from out- of- pocket payments on NCDs. Over the years from 2014 to 2017–18, the highest spike in poverty headcount was observed in Himachal Pradesh (an increase of 9.9% propelled by decline in monthly per capita expenditure and subsequent increase in out- of- pocket expenditure on NCDs). Contrarily, the state of Goa witnessed maximum shortfall (a decline of 3.0%) in the poverty headcount over the years. These inter-state heterogeneities are also depicted in Figs. 3 and 4. The higher incidence of CHE in EAG states of Bihar, Madhya Pradesh, Odisha and Uttar-Pradesh characterized by lowest per capita public spending on healthcare (Table 5, Additional file 1: Appendix) was mostly attributed to the increased burden of out- of -pocket payments amongst the poor and lower capacity to pay. However, higher incidence of CHE in developed states belonging to highest group of ETL (Goa, Himachal Pradesh, Kerala and Tamil Nadu) could be attributed to availability of more extensive health services with better physical access, thereby increasing utilization. Previous studies in the Indian context have revealed that states with lower poverty levels make higher use of public health centers, thereby rendering the care seeking more expensive in developed states. Adjusting for socio-economic correlates, the cost per hospitalization episode amongst the poor using public health centers was 51% lower than for non-poor using private health centers in India [31].

**Table 8:** State-Wise Estimates of Impoverishment due to Catastrophic Health Expenditures amongst households with NCD burden

	State Poverty Lines		HH(%) pushed under poverty due to OOPE in 2014			HH(%) pushed under poverty due to OOPE in 2017–18		
	Rural (INR)	Urban (INR)	Rural	Urban	Total	Rural	Urban	Total
Jammu & Kashmir	1044	1403	2.53	3.31	5.85	3.00	4.55	7.55
Himachal Pradesh	1067	1412	10.03	2.06	12.08	17.85	4.18	22.03
Punjab	1127	1479	10.10	8.00	18.10	11.07	7.78	18.85
Uttarakhand	1015	1408	1.98	9.90	11.88	6.83	10.00	16.83
Haryana	1128	1528	6.67	5.49	12.16	7.08	5.76	12.84
Rajasthan	1036	1406	5.55	3.91	9.45	5.61	6.07	11.68
Uttar Pradesh	890	1330	5.19	3.76	8.95	5.08	5.16	10.25
Bihar	971	1229	3.75	2.41	6.16	2.42	4.03	6.44
Sikkim	1126	1543	3.45	3.45	6.90	4.69	1.95	6.64
Arunachal Pradesh	1151	1483	0.92	1.83	2.75	3.29	1.23	4.53
Nagaland	1230	1616	2.13	3.55	5.67	4.36	4.00	8.36
Manipur	1185	1562	1.07	1.78	2.85	2.17	3.72	5.88
Mizoram	1231	1704	2.03	2.03	4.06	2.00	2.99	4.99
Tripura	936	1377	3.47	5.49	8.96	8.43	5.02	13.45
Meghalaya	1111	1524	0.81	4.84	5.65	1.04	6.77	7.81
Assam	1006	1420	3.48	4.21	7.69	4.62	5.49	10.11
West Bengal	934	1373	3.83	7.42	11.25	6.87	8.78	15.65
Jharkhand	904	1272	2.52	5.03	7.55	3.77	8.14	11.91
Odisha	876	1205	4.70	4.35	9.05	5.50	4.71	10.21
Chhatisgarh	912	1230	2.42	6.95	9.37	3.06	6.25	9.31
Madhya Pradesh	942	1340	4.67	5.22	9.89	4.15	5.81	9.97
Gujarat	1103	1507	3.79	5.80	9.59	5.72	8.76	14.48
Maharashtra	1078	1560	5.25	5.57	10.83	3.84	9.47	13.31
Andhra Pradesh	1032	1371	7.03	7.86	14.89	11.62	8.83	20.44
Karnataka	975	1373	3.83	6.96	10.79	6.18	9.20	15.38
Goa	1201	1470	9.80	6.86	16.67	8.22	5.48	13.70
Kerala	1054	1354	9.22	8.16	17.37	12.73	8.00	20.73
Tamil Nadu	1082	1380	7.28	9.59	16.87	8.48	11.60	20.08

### Determinants of catastrophic health expenditure

This section delves into socio-economic and demographic factors associated with catastrophic health expenditure computed at 10% threshold. The detailed results are tabulated in Table 9. The factors that significantly increased the odds of catastrophic health expenditure in 2014, were revealed as – demographic characteristics such as households with composition of only elderly members (odds ratio [OR] = 1.59; *P* < 0.1), socio-structural measure of households with head educated up-to primary school only (odds ratio [OR] = 1.25; P < 0.1), enabling attribute of treatment seeking from mix of public and private hospitals for various spells of ailments (odds ratio[OR] = 1.98; *P* < 0.01), seeking treatment from only private hospital for all spell of ailments (odds ratio[OR] = 3.25; *P* < 0.01), households incurring borrowings and liquidating other sources of finance to pay for treatment (odds ratio [OR] = 5.002; *p* < 0.01 and odds ratio[OR] = 1.764; *p* < 0.05 respectively) and households dwelling in poorer living conditions (odds ratio[OR] = 1.26; *P* < 0.1). Further, need- based component of households requiring hospitalization in a different state (odds ratio [OR] =1.80, *p* < 0.01) also increased the likelihood of incurring catastrophic health expenditure. Conversely; contextual factor of spatial locality i.e. households located in urban area (odds ratio [OR] = 0.80; *p* < 0. 05], households belonging to lower-middle, higher middle and high ETL states [0.442; *p* < 0.01; 0.653; *p* < 0.01 and 0.490; *p* < 0.01], enabling component of households belonging to higher income quintiles (Middle Income quintile odds ratio[OR] = 0.664; *p* < 0.01; Rich income quintiles odd ratio [OR] = 0.551; *p* < 0.01 and Richest income quintile odds ratio[OR] = 0.420; *p* < 0.01], households with insurance/financial risk protection coverage [0.680; *p* < 0.01] and household demographics such as households without the presence of elderly people [0.723; *p* < 0.01], were significantly less likely to incur catastrophic health expenditure.

**Table 9:** Logistic regression results Unravelling the Predictors of Catastrophic Health Expenditure

VARIABLES	ODDS RATIO	
	2014	2017–18
**PREDISPOSING FACTORS**		
***Demographics***		
**Age of household head**		
> 60	Ref.	Ref.
< 60	0.894 (0.124)	1.175 (0.151)
**Gender of household head**		
Male	Ref.	Ref.
Female	0.974 (0.172)	1.067 (0.177)
**Age composition of household members**		
No children or old	Ref.	Ref.
With children but no older people	0.723*** (0.0902)	0.960 (0.105)
With older people but no children	1.114 (0.153)	1.076 (0.132)
With both children and older people	0.937 (0.156)	0.848 (0.130)
Older people only	1.589* (0.390)	0.935 (0.198)
**Household gender composition**		
No men	Ref.	Ref.
Both men and women	1.256 (0.312)	0.705 (0.151)
Only men	1.037 (0.433)	0.684 (0.251)
**Household Size**		
1–3 members	Ref.	Ref.
4–6 members	1.082 (0.114)	0.973 (0.0945)
7 or more members	1.252 (0.179)	0.917 (0.125)
***Social-Structural Factors***		
**Educational status of household head**		
Illiterate or without formal schooling	Ref.	Ref.
Upto primary school	1.246* (0.156)	1.121 (0.117)
Upto middle school	1.143 (0.139)	0.930 (0.109)
Secondary to higher secondary	1.060 (0.130)	1.210* (0.122)
Graduation and above	0.964 (0.161)	0.949 (0.134)
**Principal Occupation of Household**		
Regular wage	Ref.	Ref.
Self-Employed	1.111 (0.115)	0.868 (0.0819)
Casual Labour	0.944 (0.122)	0.911 (0.102)
Others	1.130 (0.187)	0.967 (0.148)
**Social Group**		
Scheduled Castes/Scheduled Tribes	Ref.	Ref.
Other Backward Class	0.899 (0.0980)	0.996 (0.0928)
Other Groups	1.065 (0.129)	1.054 (0.103)
**ENABLING FACTORS**		
**Monthly Per Capita Expenditure Quintiles**		
Poorest	Ref.	Ref.
Poor	0.931 (0.129)	0.806* (0.103)
Middle	0.664*** (0.0972)	0.541*** (0.0683)
Rich	0.551*** (0.0809)	0.438*** (0.0612)
Richest	0.420*** (0.0675)	0.245*** (0.0349)
**Living Condition Index**		
Lowest	Ref.	Ref.
Middle	1.206 (0.146)	0.935 (0.108)
Highest	1.257* (0.157)	0.986 (0.0921)
**Whether Covered by Insurance or Pre-payment scheme**		
No	Ref.	Ref.
Yes	0.680*** (0.0628)	0.787*** (0.0633)
**Source of finance to pay for treatment**		
Household income/savings	Ref.	Ref.
Borrowings	5.002*** (0.782)	2.693*** (0.363)
Other Sources	1.764** (0.418)	0.666*** (0.0968)
**Care-seeking choices for NCD related treatment of households**		
Only Public	Ref.	Ref.
Both Public and Private	1.977*** (0.239)	2.223*** (0.228)
Only Private	3.249*** (0.309)	3.536*** (0.283)
**NEED FACTORS**		
**Hospitalization episode for NCDs over past 365 days**		
No	Ref.	Ref.
Yes	0.967 (0.0697)	1.248*** (0.0719)
**Number of household members who fell ill and sought care due to NCDs**		
One	Ref.	Ref.
Two	2.027 (0.270)	1.819 (0.157)
Three or more	3.131 (0.395)	2.886 (0.319)
**Whether member(s) of household sought inter-state hospitalization**		
No	Ref.	Ref.
Yes	1.797*** (0.313)	2.609*** (0.347)
**CONTEXTUAL FACTORS**		
**Level of epidemiological transition (ETL) of state where household is located**		
Low ETL	Ref.	Ref.
Lower-Middle ETL	0.442*** (0.0731)	0.664** (0.113)
Higher-middle ETL	0.653*** (0.0713)	0.669*** (0.0650)
High ETL	0.490*** (0.0604)	0.557*** (0.0586)
**Location**		
Rural	Ref.	Ref.
Urban	0.798**(0.0769)	0.968 (0.0877)
**Constant**	1.384 (0.452)	2.087 (0.664)
**Observations**	26,190	37,980

Standard error (S.E) in parentheses; Level of significance: *** *p* < 0.01, ** *p* < 0.05, * *p* < 0.1

However, in 2017-18; social structural characteristic of households with household heads educated upto secondary to higher- secondary level vis-à-vis illiterate/ informally educated increased the odds of incurring catastrophic health expenditure (odds ratio [OR] = 1.21; *p* < 0.1), Further, need for care, in terms of getting hospitalized for NCD in the household over the 365 days prior to the survey (odds ratio [OR] = 1.25; *p* < 0.01) had increased odds of financial catastrophe for the household. Amongst the enabling and need -based factors, in tandem with the previous findings of 2014, households seeking treatment from a mix of public and private hospitals for various spells of ailments (odds ratio [OR] = 2.22; *p* < 0.01); households seeking treatment from only private hospital for all spell of ailments (odds ratio [OR] = 3.54; p < 0.01), households borrowing money for treatment (odds ratio [OR] = 2.69; *p* < 0.0) and households having hospitalization in different states (odds ratio [OR] = 2.61; *p* < 0.01) were more likely to incur catastrophic health spending. Contrarily, households with higher income group (Middle income quintile odds ratio [OR] = 0.54; *p* < 0.01, Rich income quintiles odd ratio [OR] = 0.44; *p* < 0.01 and Richest income quintile odds ratio [OR] = 0.24; *p* < 0.01), households with financial risk protection (odds ratio [OR] = 0.79; *p* < 0.01), households residing in states grouped under advanced ETL level (Lower middle ETL group odds ratio [OR] = 0.664; *p* < 0.01,), higher middle ETL (0.669; *p* < 0.01) and high ETL (0.557; *P* < 0.01) vis-à-vis  lowest group of ETL and households financing treatment with sources other than savings and borrowings (0.666; *p* < 0.01) were associated with lower probability of having catastrophic expenditure.

## Discussion and conclusions

Our study revealed the incidence and intensity of catastrophic payments and subsequent impoverishment due to NCDs in India during the period of 2014 and 2017–18. We further investigated the socio-economic and demographic determinants influencing the catastrophic payments during the study years. The salient findings from the study are surmised as follows – a) Households were economically vulnerable to illness from NCDs with around two-third households with NCDs incurring catastrophic expenditure at 10% threshold b) Catastrophic payments were concentrated amongst the poor with further widening of inequality in incidence of catastrophic payments from 2014 to 2017–18 c) The intensity (depth) of catastrophic payments was colossal with around two-fifth and one-third of all households with NCDs spending beyond 10% catastrophic threshold in 2014 and 2017–18 respectively d) The inequalities disfavoring poor in the intensity (depth) of catastrophic payments deepened from 2014 to 2017–18 e) Level of impoverishment increased due to OOP payments on NCDs, as around one-twelfth and one-eighth  of all households with NCD burden in 2014 and 2017–18 respectively, were pushed to poverty due to healthcare expenses on treatment of NCDs f) Severity of impoverishment amongst those households that were already poor increased further by a fraction of one- fourth and one- fifth respectively in 2014 and 2017–18, connoting further deepening of poverty h) There were substantial inter-state heterogeneities in the headcount for catastrophic payments and impoverishment with states having higher burden of NCDs and economically backward status estimated to have higher levels of  CHE  and poverty- deepening impacts.

Our findings are convergent with previous literature which revealed that likelihood of incurring catastrophic payments and distressed financing in India was inordinately large for NCDs. The incidence was more exacerbated for rural residents vis-à-vis urban counterparts [32, 33], which was also found in our study (see Figs. 1 and 2, Additional file 1: Appendix). Furthermore, results were in tandem with evidence demonstrating that poor households were less able to cope with healthcare costs compared to their affluent counterparts [18, 34–36]. Studies have indicated that households positioned closer to the poverty line face much higher risk of falling into poverty trap if treatment is sought and expenditure is incurred. These findings validate the equity staircase model propounded by Tugwell et al. [37] which posits that poor face higher risks of disease and mortality; lower financial or physical access to prevention, diagnosis, and treatment; and structural challenges that diminish effectiveness of interventions.

In India, on an average, the out-of-pocket expenditure is copiously higher than the WHO estimate for developing countries which is predominantly attributed to paucity of insurance coverage and social security net. As a corollary, catastrophic payments towards healthcare precipitates into increase in impoverishment. In India, a major proportion of the OOP on NCD care was associated with hospitalization- related expenses and procedures. The rate of hospitalization amongst individuals ailing with NCDs increased from 2014 to 2017–18. In 2014, 36.3% individuals in rural areas and 43.9% individuals in urban areas suffering from chronic NCD- related conditions were hospitalized which increased by a colossal 33% in 2017–18.(Fig. 3, Additional file 1: Appendix). Concurrently, cost of hospitalization also increased over the years as average OOP on NCD- related hospitalization increased by INR 4461. However, the financial protection against hospitalization- related expenses were abysmal in both rural and urban areas. In 2014, merely 86% population in rural areas and 82% in urban areas [15] were covered under any scheme of health expenditure support. However, the coverage remained unchanged in 2017 for rural population and witnessed 1% decline among urban population [16]. Amongst the covered population, major coverage was through government- sponsored scheme of Rashtriya Swasthya Bima Yojana (RSBY) for those below poverty line and in unorganized labour. However, there were some major lacunae in this cover as indicated by an impact evaluation of the scheme which revealed that RSBY could not provide any significant financial protection for poor households [38]. The scheme excluded cover on outpatient expenditure and provided a yearly cover of just INR 30,000 per household for hospitalization expenses, impelling households to extend expenses much above the stipulated cap. Persistently high catastrophic expenditure on NCDs in India and subsequent impoverishment insinuates that schemes such as RSBY and National Health Mission were ineffective in protecting poor households from economic shocks. Thus, it is an imperative to have an augmented financial risk protection mechanism in India, particularly for the poor and the vulnerable. However, in 2018, Government of India launched a flagship scheme of Ayushman Bharat (AB-PMJAY) aimed at providing financial risk protection against health shocks to bottom 40% population of India, with a cover of INR 5 lakh per household annually for secondary and tertiary care hospitalizations which is around 16 times higher than the precursor scheme. Albeit, the outpatient expenses are not covered under AB-PMJAY which constitutes majority of OOPE in chronically ill patients suffering from NCDs.

Results elucidate that there is deepening of health insecurity in India on account of healthcare expenses which is recounted in previous studies as well. Evidence from India revealed that households are grappling with double whammy of dwindling public provisioning of healthcare and rising healthcare costs [39]. Concomitantly, there is a burgeoning of private providers in the last twenty years, compelling patients to seek care from private providers with high user fee and procure drugs and diagnostics from private outlets. During our survey years, only 22.4% households sought treatment from public providers for NCDs, whereas thrice that number sought treatment from private providers in 2014; correspondingly; in 2017–18, 27.6% households resorted to public providers and disproportionately large number (63%) sought care from private providers. Also, Indian evidence from India divulges that public providers are usually fraught with problems pertaining to quality and availability of basic amenities at healthcare centers in rural areas, thus, impelling a large proportion of ailing persons to seek treatment from expensive private health centers located in urban areas. Our survey data revealed that households which sought care in only public facilities for NCDs incurred an average OOPE of INR 1161 vis-à-vis households which solely sought care from private providers, incurred INR 2550 on an average for treatment episode in 2014. The gap in treatment costs between providers was exacerbated in 2017–18 with households paying INR 1113 to public providers and twice the amount (INR 2761) to private providers. Such inflated costs often culminate in distressed financing by households in the form of borrowings and selling of assets to cope with health shocks and catastrophic payments. Thus, it is pertinent to augment public provisioning and public subsidy on healthcare by increasing the government budget share on health. The National Health Policy, 2017 proposed to increase the central government spending on health from current level of 1.15 to 2.5% of GDP by 2025 and explicitly enunciates the goal of reducing the proportion of households incurring catastrophic health expenditure from current level by 25%, by 2025 [40]. Previously, Government of India also launched targeted National Programme for Prevention and Control of Cancer, Diabetes, Cardiovascular Disease and Stroke [41] in 2010 to give impetus to capacity of district health systems for prevention, early diagnosis, treatment and rehabilitation for these NCDs at an affordable cost. However, implementation bottlenecks and ineffective monitoring and evaluation rendered the program ineffectual in improving coverage.

Affordable access to medicines is one of the onerous factor influencing OOPE for NCDs, since the chronic ailments require treatment over a prolonged period of time. Expenditure on medicines constituted, on an average, roughly one-fifth of OOPE on NCD related hospitalization and one-third of OOPE on outpatient related expenditure. However, the affordability of medicines was highly contingent upon choice of provider, with highest CHE burden in private facilities as OOP payment on medicines was almost universally mandatory to access medicines in private facilities (Fig. 4-Fig. 16, Additional file 1: Appendix). For inpatient care, around one-fourth (24.8% in 2014 and 28% in 2017–18) of the patients received completely free medicines in public facilities located in rural areas, whereas, only 3.5 and 1.8% patients admitted in rural private facilities received free medicines in 2014 and 2017–18 respectively. The proportion of inpatients receiving completely free medicines in public facilities in urban areas was even greater than rural areas (33% in 2014 and 37.1% in 2017–18 respectively), Conversely, access to free medicine in private facilities located in urban areas for inpatients was further subjacent(3.2% in 2014 and 1.6% in 2017–18) than rural areas. The divergence in the access to free medicines between public and private facilities was even more pronounced for outpatient care. In rural areas, around one-third outpatients (33.9% in 2014 and 35.1% in 2017–18 respectively) paid full amount for medicines in public facilities, however, a colossal proportion of 90.7% in 2014 and 88.6% in 2017–18 in private rural facilities paid out of pocket price for medicines. Similarly, in urban public facilities, lesser proportion of outpatients (38.7 and 36% in 2014 and 2017–18 respectively) did not receive free medicines vis-à-vis private counterparts (91.5 and 89.5% in 2014 and 2017–18 respectively). Relatively affordable medicines in public facilities can be attributed to central government schemes like Pradhan Mantri Bharatiya Janaushadi Pariyojana (PMBJP) that explicitly aims to reduce out-of-pocket expenditure on medicines by making quality generic medicines available at affordable prices [42]. The impact evaluation of the scheme in reducing the incidence of CHE can be explored in future studies. Further, underscoring the dichotomy in the sectors, the incidence of CHE for outpatient care (Fig. 8, Additional file 1: Appendix) exhibited higher prevalence for OOP burden when treatment was sought from private providers vis-à-vis public providers,thereby indicating the need for more decisive interventions by the government in improving the quality of services in public facilities. Our multivariate analysis also conceded that households in rural areas were more likely to endure the impact of catastrophic payments despite lower OOPE on an average in rural areas (INR 1971) as compared to urban areas (INR 2698) in 2014 and subsequently, INR 2077 and INR 2729 in rural and urban areas respectively in 2017. Similar observations with respect to location were resounded in 2004–05 as well, reflecting disproportionately more impoverishment in rural areas over a protracted period of time [39]. Further, the states with higher burden of NCDs (high ETL groups) were more susceptible to catastrophic payments due to higher utilization, whereas, lower burden of catastrophic expenditure was found in states with lower levels of epidemiological transition that concurs with other studies in India [31]. A plausible explanation for lower burden in ETL groups is the prevalence of unmet need in these states as the states at the lower level of epidemiological transition are also economically backwards, thereby, impeding financial accessibility to seek care. An inverse relationship has been found between epidemiological transition ratio and socio-economic development of states [1]. India’s policies however, have been focused on health improvements in less developed Empowered Group states of Bihar, Chhattisgarh, Jharkhand, Madhya Pradesh, Odisha, Rajasthan, Uttar Pradesh; whereas, the incidence of NCDs and its associated economic burden has been substantial in developed states as well [43]. Public health expenditure as a percentage of Gross State Domestic Product (GSDP) in states with highest NCD burden (1.34, 1.68, 0.93, 0.87 and 0.74% for Goa, Himachal Pradesh, Kerala, Punjab and Tamil Nadu respectively) was revealed to be lowest across the states and much beneath the recommended 3% goal. These inter-state heterogeneities has pertinent policy ramifications and it is suggested that promulgation of state-specific policies should be done that regards the contextual variations and budgetary allocations for health sector should be revised with increased prioritization.

Our study has few limitations emanating from caveats in the survey data and methodological approach. *Firstly,* the outcome measure is non-normative as the weights placed on catastrophic payments incurred by poor and non-poor households were same and ignores the fact that opportunity cost of health spending is different between poor and non-poor households. The measures do not allow for distributional sensitivity and results should be interpreted with caution as the same threshold was used for different socio-economic groups. Assuming the diminishing marginal utility of money, beyond a fixed threshold level, the marginal utility of next amount is much higher for poorer households. Thus, it is more appropriate to consider distribution-sensitive measures of catastrophe at different levels for disparate socio-economic groups which is in tandem with vertical equity principle insinuating that higher expenditure proportions are required to designate a richer household as having experienced a catastrophic event [44]. *Secondly,* we did not consider coping-adjusted health expenditures in the study. Subsequently, the ‘hidden’ poverty due to inflation of total household expenditure by financial coping strategies and ‘transient’ impoverishment due to sacrifice of necessary consumption to temporarily pay for health is not demonstrated. Measures that ignore coping strategies not only overstate risk to current consumption and exaggerate the scale of catastrophic payments but also overlook the long-run burden of health payments [45]. One of the lacunae of this relativist approach stems from the fact that threshold used to define catastrophic payment is inevitably somewhat arbitrary with no guarantee that spending less than this ratio is not a threat to the satisfaction of basic needs. A systematic review [46] conceded that the impact that NCDs exert on households and impoverishment is likely to be underestimated since important economic domains, such as coping strategies and inclusion of marginalized and vulnerable people who do not seek healthcare due to financial reasons, are overlooked in literature. Studies have established that economically vulnerable households may unconsciously alter their perception of an illness and thus, forgo treatment altogether. This forgone treatment could lead to an exacerbation of an illness and thus, higher catastrophic expenditures, thereby, triggering another vicious cycle [47]. Literature also underscores the high prevalence of non-adherence of the treatment and disruption in the medication for NCDs due to chronic and progressive nature of disease requiring long term management, thereby, leading to deflated estimates of out-of-pocket expenditures [48, 49]. *Thirdly,* the disaggregated impact of price of health services and quantity of services used on catastrophic payments was not disentangled as these two components are intertwined with each other. A given level of out- of- pocket spending could be a consequence of  low prices or high use or combination of both. *Fourthly,* the expenditure data used in study is self-reported that is amenable to recall bias and is not verified by other sources. A study investigating OOP expenses for diabetes patients in a tertiary care setting, highlighted that social-desirability bias led to patients overestimating their adherence to the prescription course for medications, thereby, leading to an information bias on OOP-related expenses [50]. The absence of a validating exercise in the survey design renders our study susceptible to social-desirability bias as well. Fifthly, the self-reporting of monthly household consumer expenditure without a parallel validation during the survey poses challenges in terms of underestimation of household incomes. Winter (2004) demonstrated in a controlled survey experiment that a one-shot open-ended question on household consumption yielded significantly lower estimates of consumption than a disaggregated question with multiple categories [51]. Hence, it is pertinent to note that more refined consumption expenditure estimates can be obtained from National Sample Survey (NSS) Consumption Expenditure Survey (CES) enabling estimation of capacity to pay and further studies should be conducted to ascertain sensitivity of impoverishment measures utilizing health survey and consumption expenditure survey. *Finally*, the disruption in living standards due to catastrophic payments should be ascertained with longitudinal data, however, in the absence of such data, we have used repeated cross- sectional in our study. Despite these limitations, the evidence generated by our study has important policy implications in India and other resource-constrained nations undergoing demographic and economic transition with high increasing burden of NCDs.

## Supplementary Information

**Additional file 1.**

## Data Availability

The datasets generated/or analyzed during the current study are available from Ministry of Statistics and Programme Implementation (MOSPI), Government of India upon request or can be downloaded from *https://mospi.gov.in/web/mospi/download-tables-data*
