# Supplementary material for: Assessing the household economic burden of non-communicable diseases in India: evidence from repeated cross-sectional surveys
Source: BMC Public Health. 2021 May 7;21:881. doi: 10.1186/s12889-021-10828-3 (PMC8106177; doi:10.1186/s12889-021-10828-3)
Supplement: Supplementary file 1 — Additional file 1. [file 12889_2021_10828_MOESM1_ESM.pdf]

APPENDIX

Table 1 : State-Wise Estimates for Incidence of Catastrophic Health Expenditure on Treatment of Non-Communicable Diseases in 2014								
	Households incurring Catastrophic health expenditure		Mean household monthly consumption expenditure (INR)			Mean household monthly Out-of-pocket expenditure (INR)		
State	%	CI	INR	LCI	UCI	INR	LCI	UCI
Jammu & Kashmir	77.9	[66.97-85.97]	4976.275	4159.802	5792.747	2179.83	1635.516	2724.143
Himachal Pradesh	71.4	[60.2-80.48]	5334.65	4356.199	6313.101	2227.205	1686.969	2767.441
Punjab	74.54	[66.01-81.53]	7967.3	6967.19	8967.41	2498.09	2064.591	2931.588
Chandigarh	43.5	[15.9-75.82]	9114.893	5985.556	12244.23	1869.227	623.0289	3115.425
Uttarakhand	77.61	[59.34-89.17]	3798.508	3235.486	4361.529	3041.072	1159.239	4922.906
Haryana	65.74	[54.36-75.56]	6693.706	5598.91	7788.502	2973.294	2220.899	3725.688
Delhi	62.19	[46.7-75.53]	10288.64	8338.205	12239.08	2908.01	2220.929	3595.092
Rajasthan	66.22	[59.86-72.04]	5291.432	4710.745	5872.119	2121.784	1653.888	2589.68
Uttar Pradesh	76.68	[72.5-80.4]	4461.839	4125.323	4798.354	2628.83	2210.148	3047.512
Bihar	74.21	[64.67-81.9]	3273.246	3014.407	3532.084	2675.373	1508.792	3841.954
Sikkim	48.52	[26.72-70.9]	4673.074	3547.935	5798.213	1244.965	506.4329	1983.497
Arunachal Pradesh	69.99	[50.82-84.03]	2971.419	2321.11	3621.727	1265.155	731.1024	1799.207
Nagaland	39.76	[20.02-63.52]	5873.266	4994.861	6751.672	1109.359	447.1872	1771.531
Manipur	92.29	[86.7-95.65]	3814.704	3267.27	4362.138	1811.209	1247.239	2375.179
Mizoram	41.22	[25.41-59.07]	7351.085	5953.885	8748.285	1066.526	651.0908	1481.962
Tripura	59.44	[46.74-70.99]	4060.073	3510.59	4609.557	4207.825	-1129.875	9545.526
Meghalaya	44.27	[22.72-68.21]	4373.134	3746.276	4999.992	780.5476	422.5146	1138.581
Assam	73.07	[61.71-82.04]	4184.056	3278.265	5089.847	2254.041	112.508	4395.575
West Bengal	69.96	[65.43-74.12]	4574.344	4220.723	4927.966	1905.387	1629.016	2181.757
Jharkhand	61.69	[48.41-73.43]	3938.37	3256.662	4620.079	1846.098	216.1834	3476.014
Odisha	84.92	[79.64-89.03]	3305.642	3018.43	3592.855	2110.88	1377.897	2843.863
Chhattisgarh	79.05	[65.48-88.25]	3688.225	2723.518	4652.933	3557.401	551.0773	6563.725
Madhya Pradesh	74.68	[67.67-80.61]	4076.342	3647.837	4504.847	2115.564	1762.427	2468.701
Gujarat	52.52	[46.22-58.75]	6108.612	5520.889	6696.334	1632.945	1304.933	1960.958
Daman & Diu	20.68	[3.72-63.72]	12225.22	9525.768	14924.66	1647.773	-126.2468	3421.794
Dadra & Nagar Haveli	25.39	[12.36-45.07]	6571.713	4704.194	8439.231	687.5409	279.8634	1095.219
Maharashtra	72.11	[66.98-76.72]	5607.82	5198.632	6017.008	2359.154	2024.453	2693.856
Andhra Pradesh (Undivided)	66.54	[61.2-71.48]	5240.864	4896.428	5585.299	2268.721	1869.38	2668.062
Karnataka	72.9	[65.96-78.88]	4702.553	4323.321	5081.785	1985.087	1683.126	2287.047
Goa	68.37	[52.43-80.92]	7079.753	6173.14	7986.366	2087.889	1101.582	3074.196

Lakshadweep	35.94	[17.47-59.79]	5016.815	4106.994	5926.636	1362.2	458.9754	2265.425
Kerala	60.62	[56.34-64.75]	6646.088	6162.336	7129.839	2638.425	2116.029	3160.82
Tamil Nadu	56.79	[51.21-62.2]	5290.024	4918.557	5661.49	1935.895	1596.566	2275.224
Puducherry	44.8	[30.5-60.02]	6303.696	5164.382	7443.01	2397.37	791.6104	4003.13
Andaman & Nicobar Islands	19.8	[8.86-38.53]	6847.549	5161.785	8533.313	3232.403	-2080.058	8544.864

Table 2: State-Wise Estimates for Incidence of Catastrophic Health Expenditure on Treatment of Non-Communicable Diseases in 2017-18

	**Households incurring Catastrophic health expenditure**		**Mean household monthly consumption expenditure (INR)**			**Mean household monthly Out-of-pocket expenditure (INR)**		
	**%**	**CI**	**INR**	**LCI**	**UCI**	**INR**	**LCI**	**UCI**
Jammu & Kashmir	50.98	[43.42,58.5]	6679.862	6193.436	7166.287	1140.291	948.55	1332.033
Himachal Pradesh	62.82	[54.43,70.49]	6593.264	6100.884	7085.644	3202.222	2131.264	4273.18
Punjab	55.31	[48.48,61.94]	9693.035	8994.675	10391.39	2459.512	2064.528	2854.495
Chandigarh	58.31	[37.82,76.28]	15729.1	11941.51	19516.68	4097.909	953.8036	7242.014
Uttarakhand	66.39	[51.5,78.61]	9534.863	4986.816	14082.91	2537.433	1904.567	3170.298
Haryana	65.54	[55.12,74.64]	8711.875	7884.529	9539.22	2503.377	2045.594	2961.159
Delhi	51.84	[36.48,66.85]	11897.09	10147.56	13646.61	2875.036	1558.759	4191.313
Rajasthan	62.18	[56.08,67.91]	6681.653	6240.717	7122.59	2805.378	2106.577	3504.179
Uttar Pradesh	76.36	[72.48,79.85]	5184.867	4939.211	5430.524	3161.915	2749.364	3574.465
Bihar	66.59	[55.37,76.2]	4383.306	3854.44	4912.171	1615.983	1208.376	2023.59
Sikkim	56.52	[40.71,71.11]	6088.487	5610.917	6566.057	1369.143	930.2425	1808.044
Arunachal Pradesh	76.96	[67.53,84.29]	5278.24	4118.053	6438.427	3306.507	1872.078	4740.936
Nagaland	73.89	[59.35,84.57]	7240.953	5545.636	8936.271	1527.29	1058.469	1996.112
Manipur	83.44	[76.95,88.38]	5879.566	5183.679	6575.453	3431.197	2426.855	4435.539
Mizoram	47.99	[35.51,60.72]	9234.147	7687.256	10781.04	1663.845	1174.414	2153.276
Tripura	60.72	[52.24,68.59]	6178.453	5496.232	6860.674	3390.702	1347.451	5433.952
Meghalaya	18.15	[12,26.51]	6885.259	6119.432	7651.085	765.6112	438.9872	1092.235

Assam	65.49	[55.52,74.27]	5461.596	4789.602	6133.59	2784.65	1658.841	3910.459
West Bengal	62.29	[58.22,66.2]	6176.692	5836.6	6516.784	2067.806	1674.367	2461.246
Jharkhand	79.65	[71.41,85.99]	5238.922	4641.385	5836.46	2543.605	1940.068	3147.142
Odisha	81.15	[75.85,85.51]	4165.324	3709.251	4621.397	1670.497	1484.096	1856.898
Chhattisgarh	63.83	[52.35,73.92]	4786.686	4122.47	5450.902	2453.375	1151.572	3755.178
Madhya Pradesh	64.94	[54.75,73.93]	5936.58	5268.882	6604.278	2632.217	2070.036	3194.397
Gujarat	55.32	[47.87,62.54]	8691.946	7953.146	9430.746	1762.826	1522.401	2003.251
Daman & Diu	94.52	[64.03,99.41]	7502.865	7192.1	7813.629	1893.124	1657.977	2128.27
Dadra & Nagar Haveli	33.38	[20.88,48.75]	7096.98	5702.132	8491.829	1432.778	585.9701	2279.585
Maharashtra	59.07	[54.57,63.42]	8725.457	7982.988	9467.927	2371.41	2133.969	2608.851
Andhra Pradesh (Undivided)	63.31	[58.58,67.8]	6168.539	5762.963	6574.116	1843.764	1635.412	2052.116
Karnataka	71.69	[64.92,77.6]	7672.076	6808.629	8535.523	2150.266	1872.328	2428.203
Goa	59.59	[41.73,75.22]	8500.606	7740.801	9260.411	1886.363	1223.97	2548.756
Lakshadweep	24.99	[16.46,36.05]	8766.724	7677.781	9855.667	1571.259	783.9042	2358.614
Kerala	61.22	[57.82,64.51]	7829.892	7520.779	8139.004	2638.033	2292.411	2983.655
Tamil Nadu	48.17	[43.02,53.35]	6233.033	5874.765	6591.3	2024.409	1534.523	2514.294
Puducherry	32.81	[17.67,52.64]	8101.416	7019.396	9183.436	3045.501	1025.652	5065.35
Andaman & Nicobar Islands	24.08	[14.88,36.54]	10995.2	9761.741	12228.66	2286.126	871.7809	3700.471

Table 3: State-wise estimates of Impoverishment due to healthcare payments on Non-Communicable Diseases in 2014										
	State Poverty Lines		HH with NCD burden	HH impoverished before health payments			HH impoverished after health payments			HH pushed under poverty due to OOPE
	RPL – Rural (INR)	RPL – Urban (INR)	Total Number of Households	Total	Rural	Urban	Total	Rural	Urban	(In %)
Jammu & Kashmir	1044	1403	513	440	255	185	470	268	202	5.85
Himachal Pradesh	1067	1412	389	270	219	51	317	258	59	12.08
Punjab	1127	1479	812	494	275	219	641	357	284	18.10
Uttarakhand	1015	1408	202	159	84	75	183	88	95	11.88
Haryana	1128	1528	510	382	210	172	444	244	200	12.16
Rajasthan	1036	1406	1100	887	483	404	991	544	447	9.45
Uttar Pradesh	890	1330	2949	2426	1496	930	2690	1649	1041	8.95
Bihar	971	1229	1120	1001	671	330	1070	713	357	6.16
Sikkim	1126	1543	145	110	74	36	120	79	41	6.90
Arunachal Pradesh	1151	1483	109	102	67	35	105	68	37	2.75
Nagaland	1230	1616	141	119	73	46	127	76	51	5.67
Manipur	1185	1562	281	265	139	126	273	142	131	2.85
Mizoram	1231	1704	197	144	82	62	152	86	66	4.06
Tripura	936	1377	346	248	140	108	279	152	127	8.96
Meghalaya	1111	1524	124	108	64	44	115	65	50	5.65
Assam	1006	1420	546	475	338	137	517	357	160	7.69
West Bengal	934	1373	2640	1973	1065	908	2270	1166	1104	11.25
Jharkhand	904	1272	477	399	189	210	435	201	234	7.55
Odisha	876	1205	873	725	508	217	804	549	255	9.05
Chhatisgarh	912	1230	331	276	168	108	307	176	131	9.37
Madhya Pradesh	942	1340	1284	1036	534	502	1163	594	569	9.89
Gujarat	1103	1507	1241	940	527	413	1059	574	485	9.59
Maharashtra	1078	1560	2189	1662	898	764	1899	1013	886	10.83
Andhra Pradesh	1032	1371	2049	1428	751	677	1733	895	838	14.89
Karnataka	975	1373	1409	1119	596	523	1271	650	621	10.79
Goa	1201	1470	102	65	30	35	82	40	42	16.67
Kerala	1054	1354	1790	999	444	555	1310	609	701	17.37
Tamil Nadu	1082	1380	2075	1369	674	695	1719	825	894	16.87

Table 4: State-wise estimates of Impoverishment due to Healthcare Payments on Non-Communicable Diseases in 2017-18										
	**State Poverty Lines**		**HH with NCD burden**	**HH impoverished before health payments**			**HH impoverished after health payments**			**HH pushed under poverty due to OOPE**
	**RPL – Rural (INR)**	**RPL – Urban (INR)**	**Total Number of Households**	**Total**	**Rural**	**Urban**	**Total**	**Rural**	**Urban**	**(In %)**
Jammu & Kashmir	1044	1403	1232	915	513	402	1008	550	458	7.55
Himachal Pradesh	1067	1412	885	463	408	55	658	566	92	22.03
Punjab	1127	1479	1337	657	362	295	909	510	399	18.85
Uttarakhand	1015	1408	410	257	151	106	326	179	147	16.83
Haryana	1128	1528	989	638	375	263	765	445	320	12.84
Rajasthan	1036	1406	1729	1246	828	418	1448	925	523	11.68
Uttar Pradesh	890	1330	3737	2955	1758	1197	3338	1948	1390	10.25
Bihar	971	1229	1242	1085	721	364	1165	751	414	6.44
Sikkim	1126	1543	256	194	167	27	211	179	32	6.64
Arunachal Pradesh	1151	1483	243	207	139	68	218	147	71	4.53
Nagaland	1230	1616	275	201	131	70	224	143	81	8.36
Manipur	1185	1562	646	571	296	275	609	310	299	5.88
Mizoram	1231	1704	401	217	114	103	237	122	115	4.99
Tripura	936	1377	498	291	195	96	358	237	121	13.45
Meghalaya	1111	1524	192	133	93	40	148	95	53	7.81
Assam	1006	1420	910	714	519	195	806	561	245	10.11
West Bengal	934	1373	3304	2070	1265	805	2587	1492	1095	15.65
Jharkhand	904	1272	823	606	375	231	704	406	298	11.91
Odisha	876	1205	1508	1145	879	266	1299	962	337	10.21
Chhatisgarh	912	1230	752	596	384	212	666	407	259	9.31
Madhya Pradesh	942	1340	1565	1182	635	547	1338	700	638	9.97
Gujarat	1103	1507	1347	828	453	375	1023	530	493	14.48
Maharashtra	1078	1560	3388	2155	1109	1046	2606	1239	1367	13.31
Andhra Pradesh	1032	1371	3194	1744	1142	602	2397	1513	884	20.44
Karnataka	975	1373	1391	840	521	319	1054	607	447	15.38
Goa	1201	1470	219	106	35	71	136	53	83	13.70
Kerala	1054	1354	2875	1350	676	674	1946	1042	904	20.73
Tamil Nadu	1082	1380	2371	1335	830	505	1811	1031	780	20.08

Table 5: Incidence of Catastrophic Health Expenditure, Per capita health expenditure and State health expenditure as % of GSDP across states at various groups of Epidemiological transition level (ETL)

	State/UT	Incidence of CHE (2014)	Incidence of CHE (2017-18)	Per Capita Health Expenditure	Health Expenditure as a % of GSDP
Lowest ETL	Assam	73.07	65.49	1546	2.21%
	Bihar	74.21	66.59	491	1.33%
	Chhattisgarh	79.05	63.83	1354	1.33%
	Jharkhand	61.69	79.65	866	1.25%
	Madhya Pradesh	74.68	64.94	716	1.04%
	Odisha	84.92	81.15	927	1.19%
	Rajasthan	66.22	62.18	1360	1.44%
	Uttar Pradesh	76.68	76.36	733	1.42%
	Meghalaya	44.27	18.15	2223	2.40%
Lower-middle ETL	Gujarat	52.52	55.32	1189	0.72%
	Uttarakhand	77.61	66.39	1765	1.06%
	Arunachal Pradesh	69.99	76.96	5177	3.29%
	Manipur	92.29	83.44	2061	2.79%
	Mizoram	41.22	47.99	5862	4.20%
	Nagaland	39.76	73.89	2450	2.97%
	Sikkim	48.52	56.52	5126	1.81%
	Tripura	59.44	60.72	2183	2.41%
Higher-middle ETL	Andhra Pradesh	66.54	63.31	1013	0.82%
	Delhi	62.19	51.84	1992	0.76%
	Haryana	65.74	65.54	1119	0.63%

	Jammu & Kashmir	77.9	50.98	2359	2.46%
	Karnataka	72.9	71.69	1124	0.69%
	Maharashtra	72.11	59.07	1011	0.60%
	Telangana			1322	0.82%
	West Bengal	69.96	62.49	778	n.a.
	Andaman & Nicobar	19.8	24.08	6201	5.23%
	Chandigarh	43.5	58.31	2224	1.32%
	Dadra & Nagar Haveli	25.39	33.38	2451	n.a.
	Daman & Diu	20.68	94.52	2073	n.a.
	Lakshadweep	35.94	24.99	6018	n.a.
	Puducherry	44.8	32.81	3340	2.13%
Highest ETL	Goa	68.37	59.59	3643	1.34%
	Himachal Pradesh	71.4	62.82	2667	1.68%
	Kerala	60.62	61.22	1463	0.93%
	Punjab	74.54	55.31	1173	0.87%
	Tamil Nadu	56.79	48.17	1235	0.74%

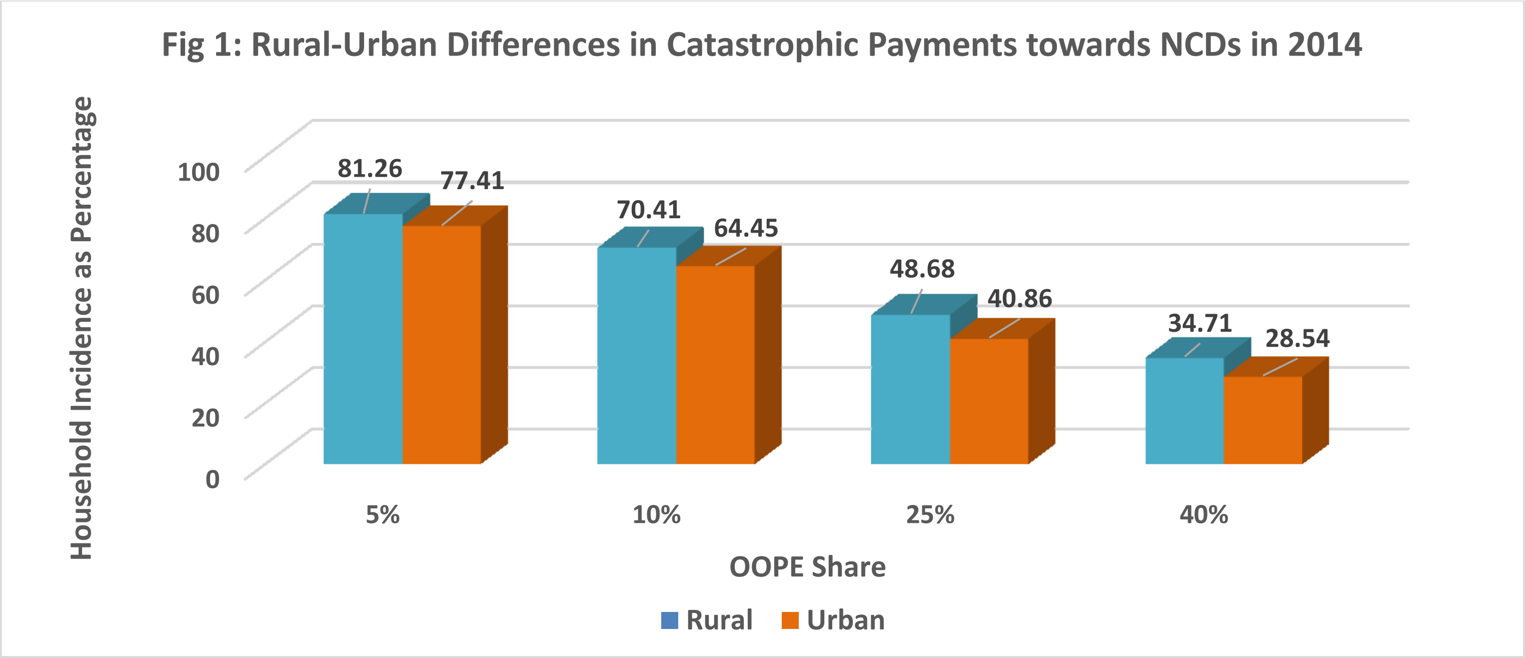
Fig 1: Rural-Urban Differences in Catastrophic Payments towards NCDs in 2014
Household Incidence as Percentage
100
80
60
40
20
0
81.26
77.41
70.41
64.45
48.68
40.86
34.71
28.54
5%
10%
25%
40%
OOPE Share
Rural
Urban

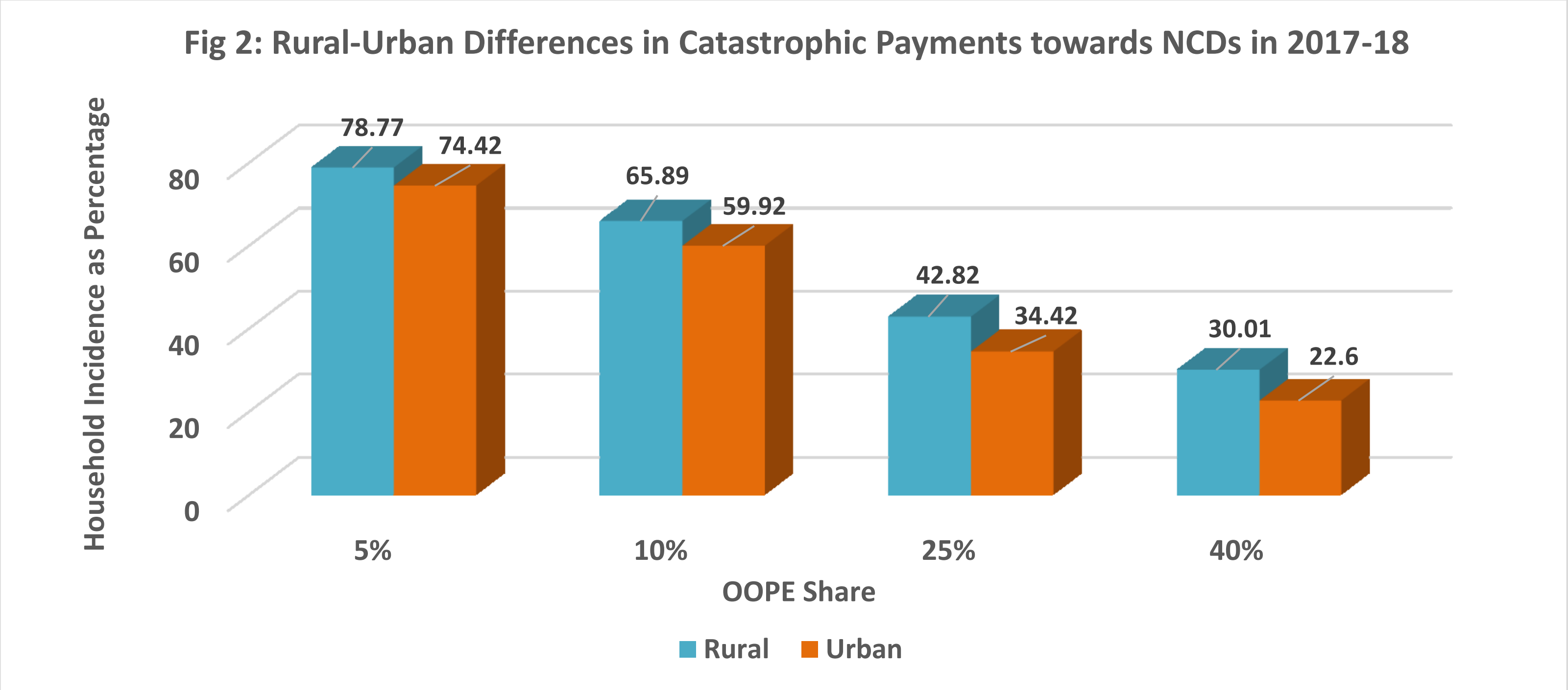
Fig 2: Rural-Urban Differences in Catastrophic Payments towards NCDs in 2017-18
Household Incidence as Percentage
0
20
40
60
80
78.77
74.42
65.89
59.92
42.82
34.42
30.01
22.6
5%
10%
25%
40%
OOPE Share
Rural
Urban

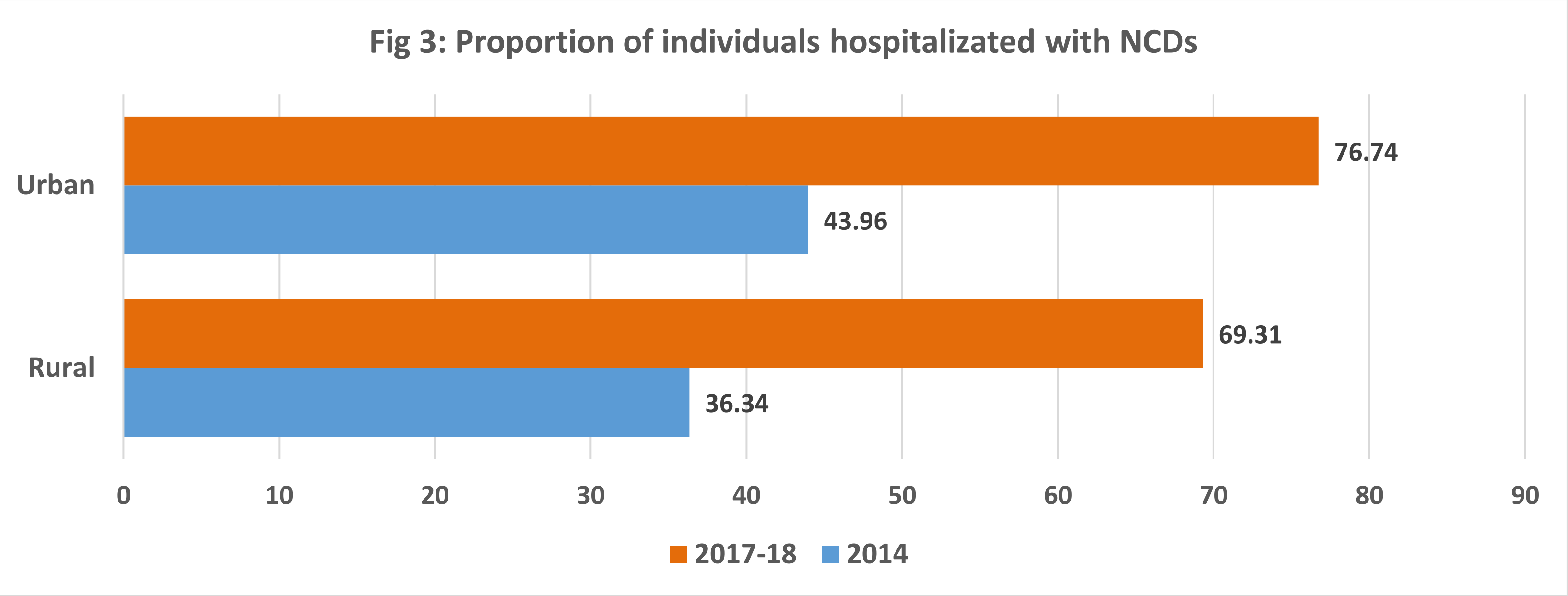
Fig 3: Proportion of individuals hospitalizated with NCDs
Urban
Rural
76.74
43.96
69.31
36.34
0
10
20
30
40
50
60
70
80
90
2017-18
2014

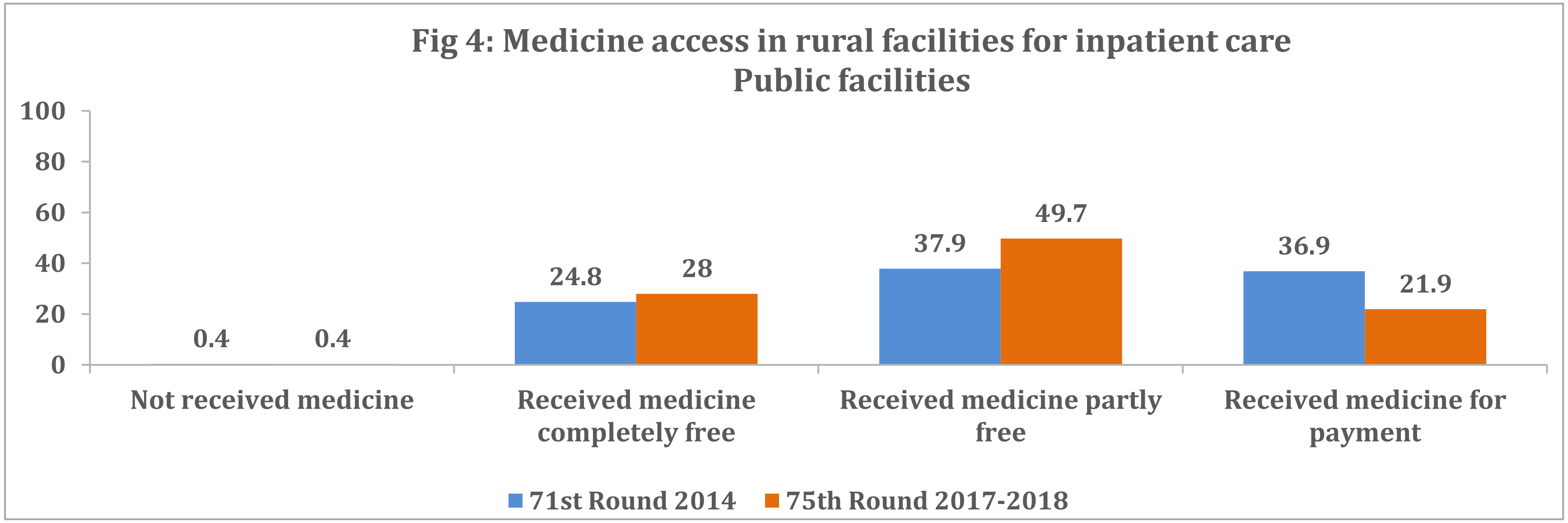

Fig 4: Medicine access in rural facilities for inpatient care
Public facilities
100
80
60
40
20
0
0.4
0.4
24.8
28
37.9
49.7
36.9
21.9
Not received medicine
Received medicine completely free
Received medicine partly free
Received medicine for payment
71st Round 2014
75th Round 2017-2018

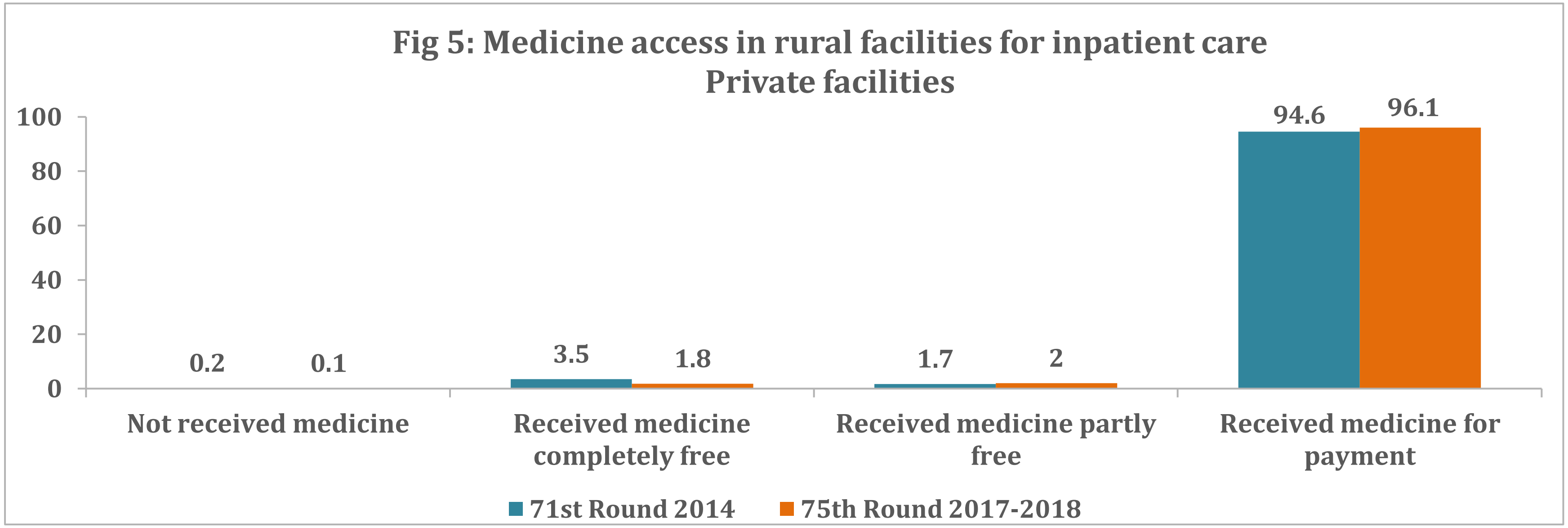

Fig 5: Medicine access in rural facilities for inpatient care
Private facilities
100
80
60
40
20
0
0.2
0.1
3.5
1.8
1.7
2
94.6
96.1
Not received medicine
Received medicine completely free
Received medicine partly free
Received medicine for payment
71st Round 2014
75th Round 2017-2018

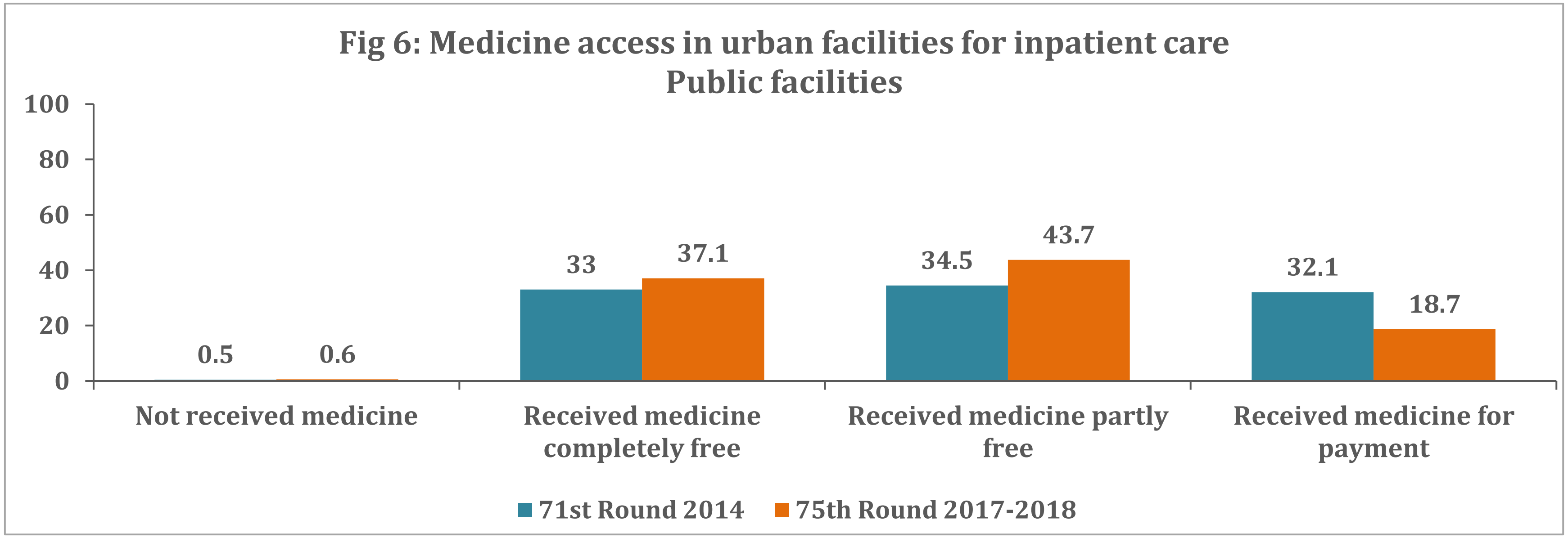
Fig 6: Medicine access in urban facilities for inpatient care
Public facilities
100
80
60
40
20
0
0.5
0.6
33
37.1
34.5
43.7
32.1
18.7
Not received medicine
Received medicine completely free
Received medicine partly free
Received medicine for payment
71st Round 2014
75th Round 2017-2018

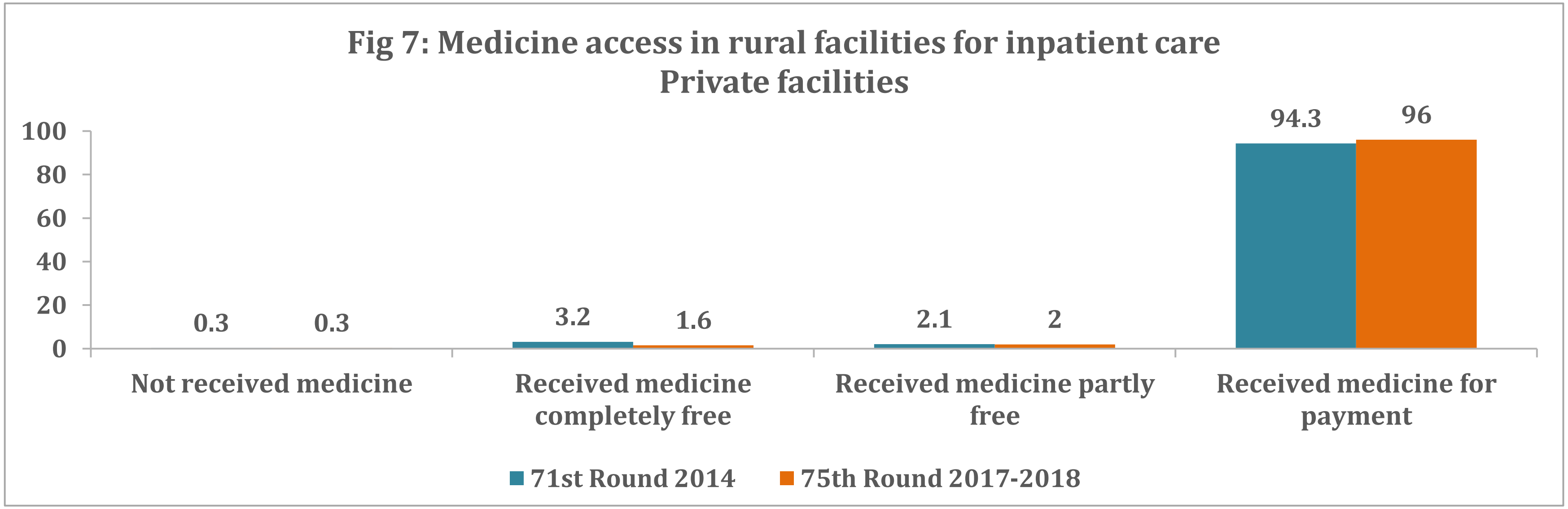
Fig 7: Medicine access in rural facilities for inpatient care
Private facilities
100
80
60
40
20
0
0.3
0.3
3.2
1.6
2.1
2
94.3
96
Not received medicine
Received medicine completely free
Received medicine partly free
Received medicine for payment
71st Round 2014
75th Round 2017-2018

Fig 8: Incidence of catastrophic health expenditure in outpatient care 2017-18

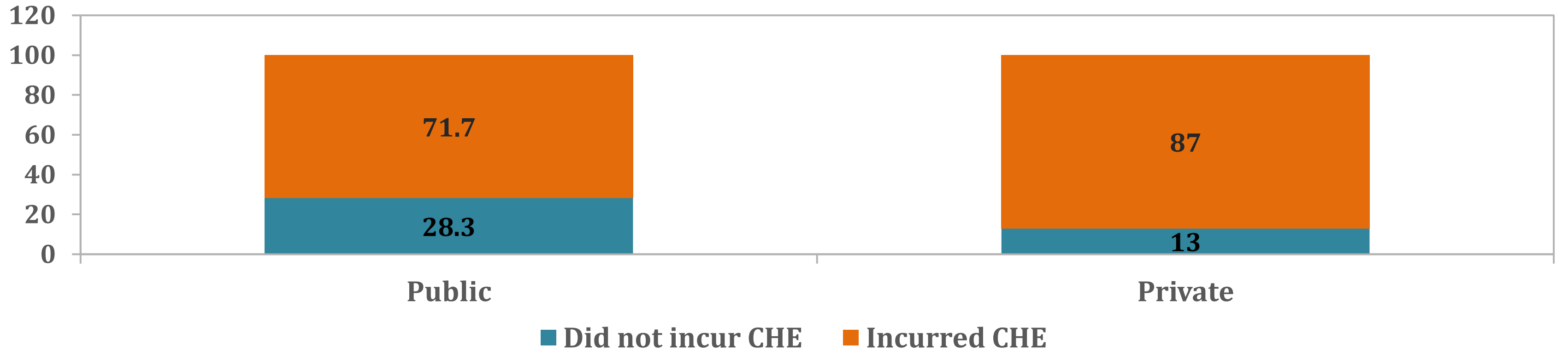

Fig 9: Access to Non-AYUSH medicines in rural facilities for outpatient care Public facilities

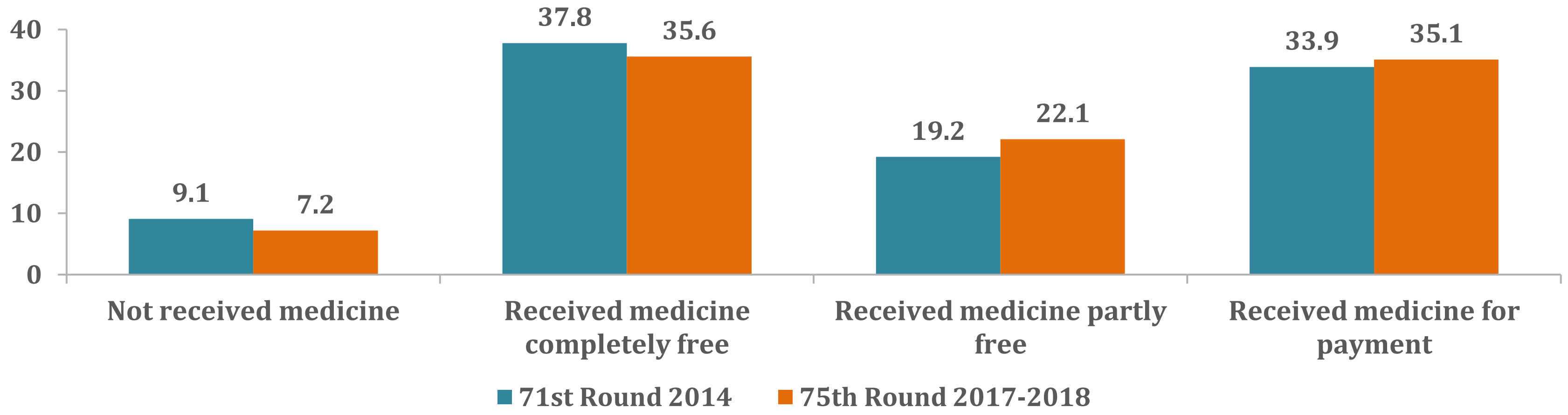

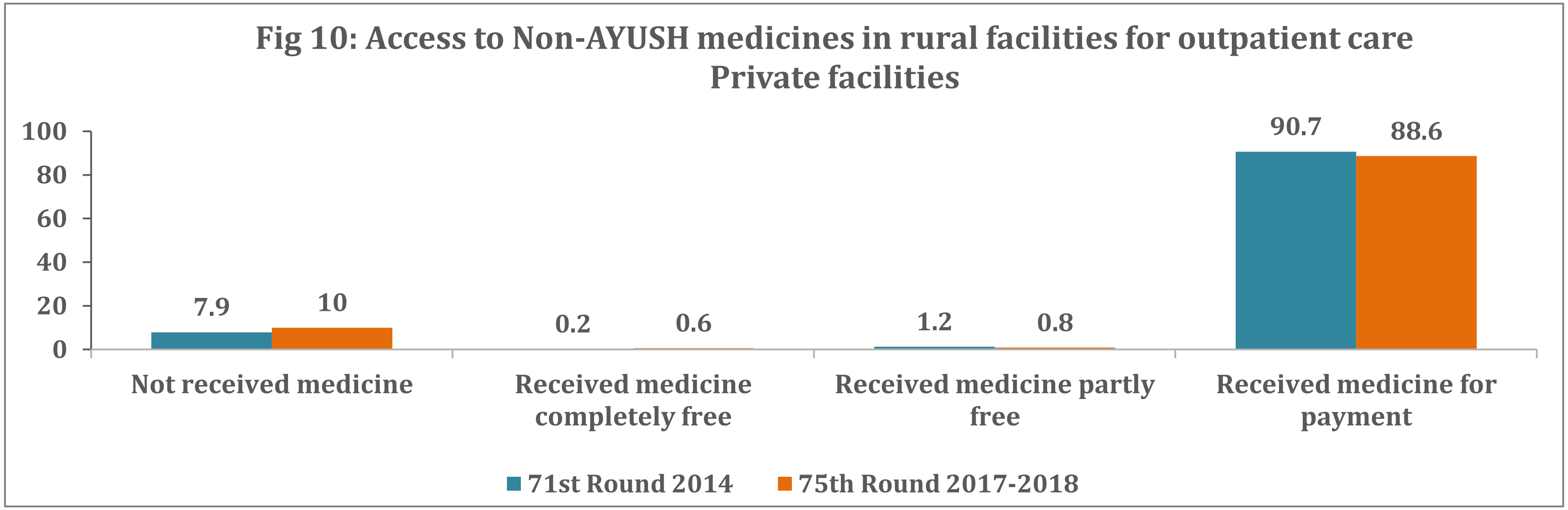
Fig 10: Access to Non-AYUSH medicines in rural facilities for outpatient care
Private facilities
100
80
60
40
20
0
7.9
10
0.2
0.6
1.2
0.8
90.7
88.6
Not received medicine
Received medicine completely free
Received medicine partly free
Received medicine for payment
71st Round 2014
75th Round 2017-2018

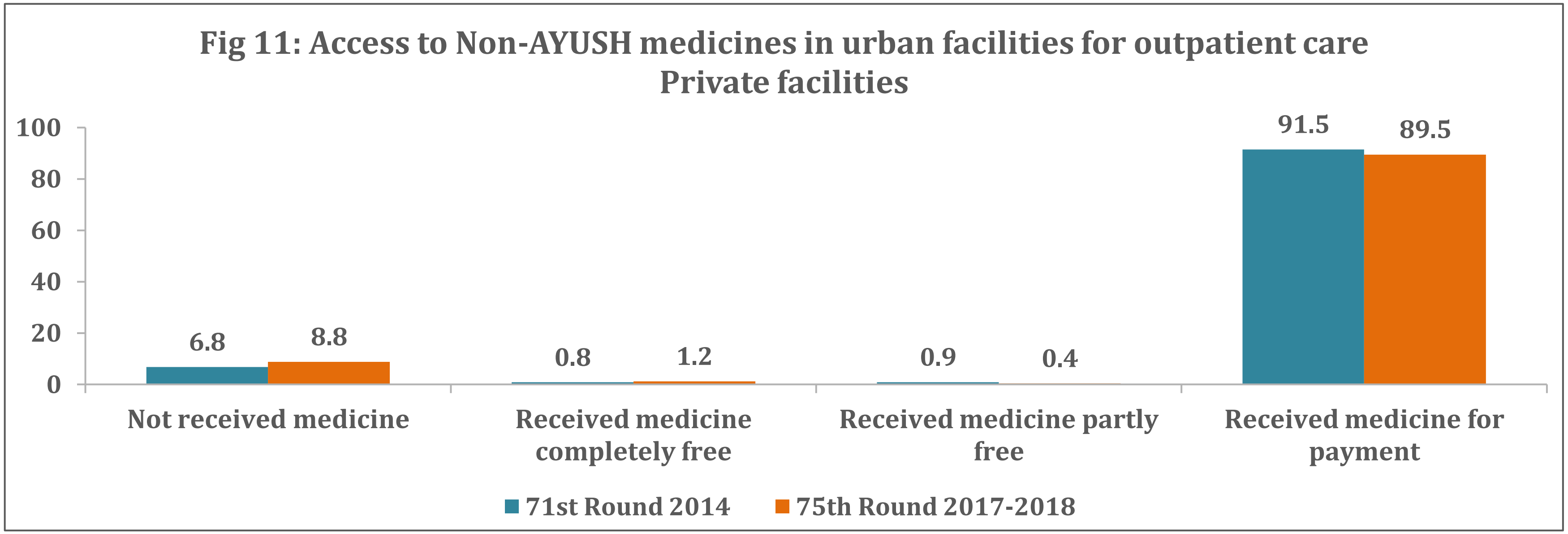
Fig 11: Access to Non-AYUSH medicines in urban facilities for outpatient care
Private facilities
100
80
60
40
20
0
6.8
8.8
0.8
1.2
0.9
0.4
91.5
89.5
Not received medicine
Received medicine completely free
Received medicine partly free
Received medicine for payment
71st Round 2014
75th Round 2017-2018

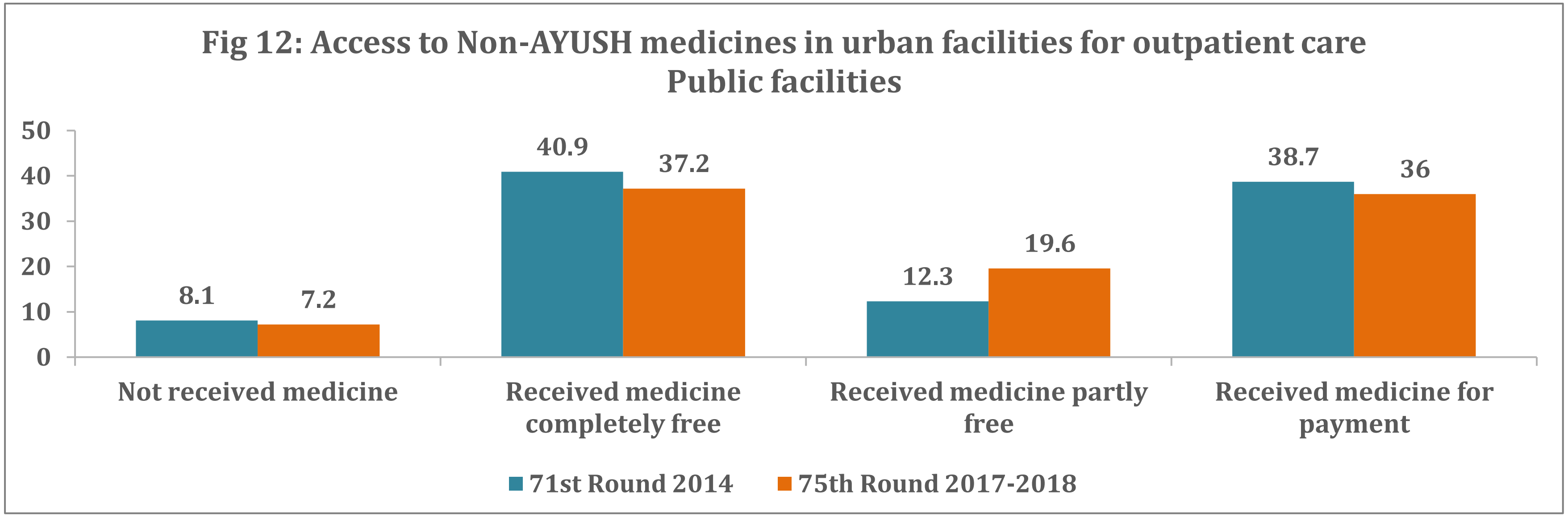

Fig 12: Access to Non-AYUSH medicines in urban facilities for outpatient care
Public facilities
50
40
30
20
10
0
8.1
7.2
40.9
37.2
12.3
19.6
38.7
36
Not received medicine
Received medicine completely free
Received medicine partly free
Received medicine for payment
71st Round 2014
75th Round 2017-2018

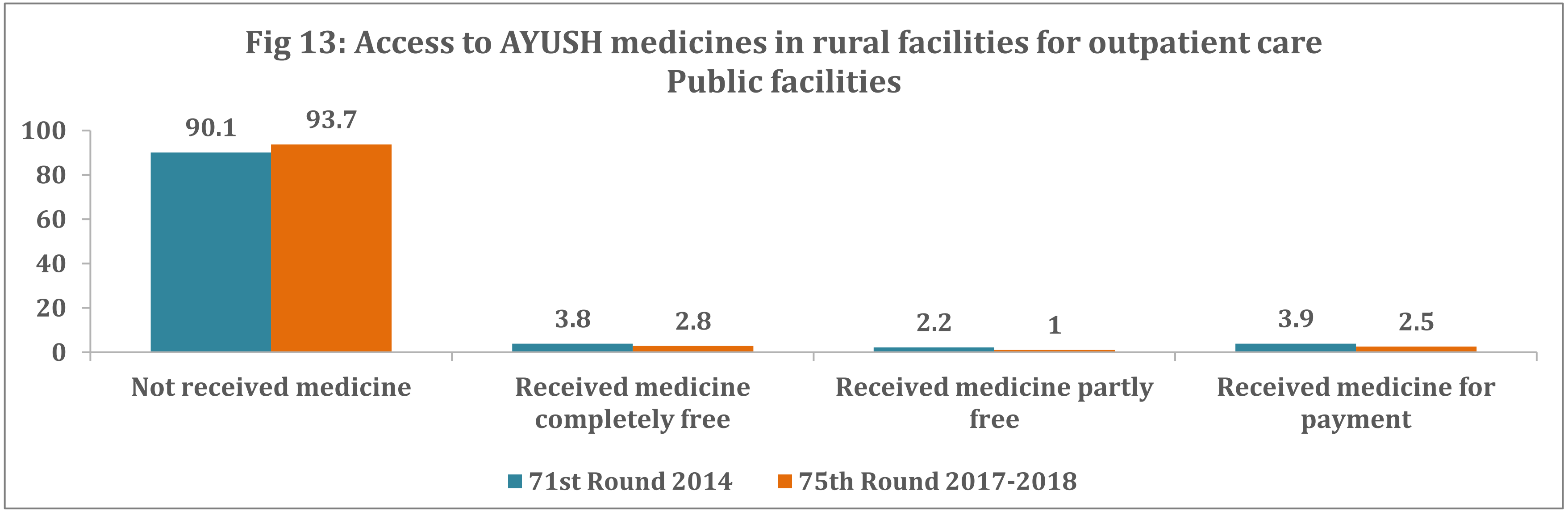

Fig 13: Access to AYUSH medicines in rural facilities for outpatient care
Public facilities
100
80
60
40
20
0
90.1
93.7
3.8
2.8
2.2
1
3.9
2.5
Not received medicine
Received medicine completely free
Received medicine partly free
Received medicine for payment
71st Round 2014
75th Round 2017-2018

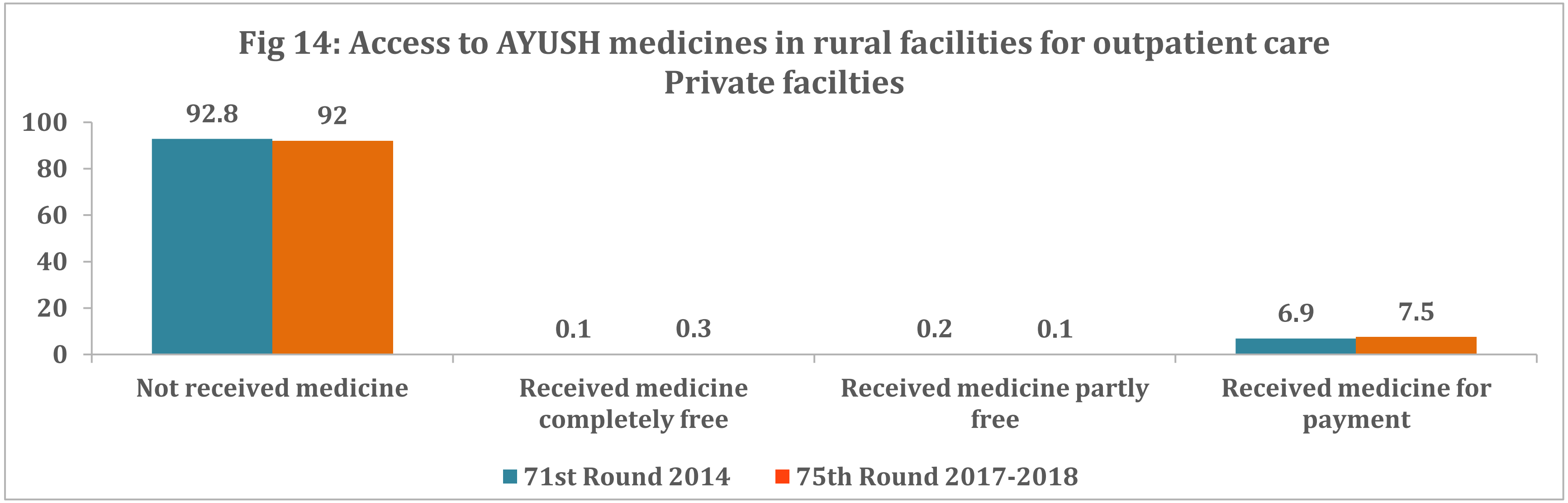
Fig 14: Access to AYUSH medicines in rural facilities for outpatient care
Private facilties
100
80
60
40
20
0
92.8
92
0.1
0.3
0.2
0.1
6.9
7.5
Not received medicine
Received medicine completely free
Received medicine partly free
Received medicine for payment
71st Round 2014
75th Round 2017-2018

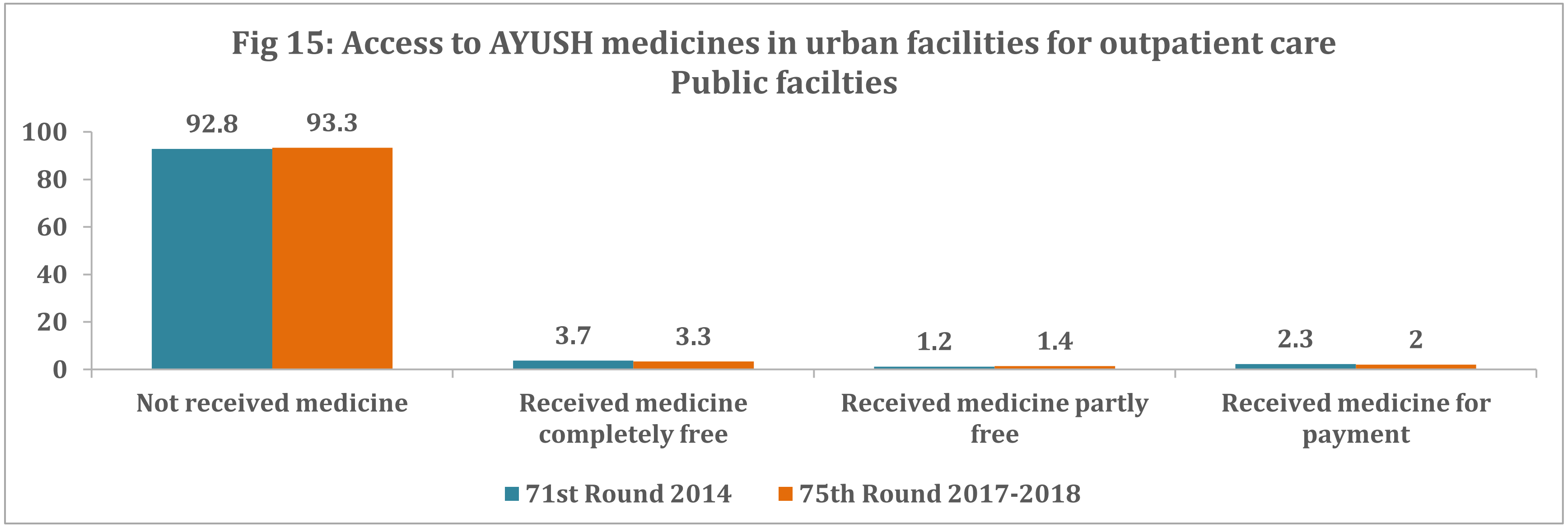
Fig 15: Access to AYUSH medicines in urban facilities for outpatient care
Public facilties
100
80
60
40
20
0
92.8
93.3
3.7
3.3
1.2
1.4
2.3
2
Not received medicine
Received medicine completely free
Received medicine partly free
Received medicine for payment
71st Round 2014
75th Round 2017-2018

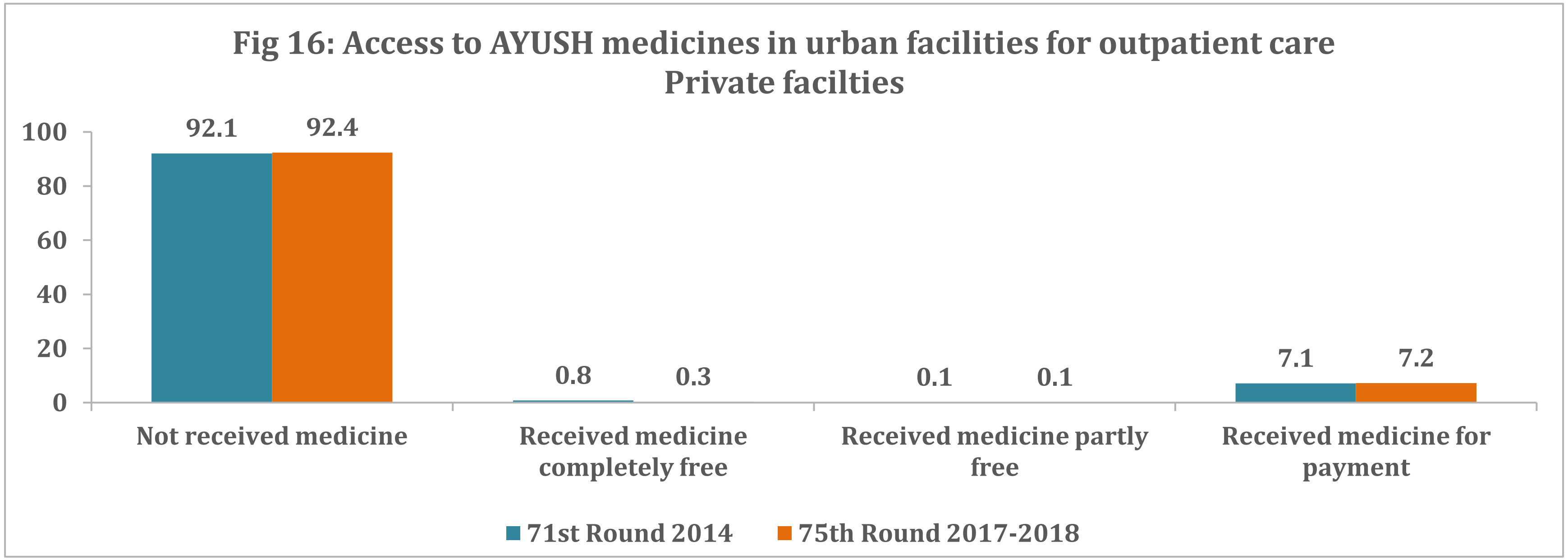
Fig 16: Access to AYUSH medicines in urban facilities for outpatient care
Private facilties
100
80
60
40
20
0
92.1
92.4
0.8
0.3
0.1
0.1
7.1
7.2
Not received medicine
Received medicine completely free
Received medicine partly free
Received medicine for payment
71st Round 2014
75th Round 2017-2018
